# Phosphorylation of the HIV-1 capsid by MELK triggers uncoating to promote viral cDNA synthesis

**DOI:** 10.1371/journal.ppat.1006441

**Published:** 2017-07-06

**Authors:** Hiroaki Takeuchi, Hideki Saito, Takeshi Noda, Tadashi Miyamoto, Tomokazu Yoshinaga, Kazutaka Terahara, Hiroshi Ishii, Yasuko Tsunetsugu-Yokota, Shoji Yamaoka

**Affiliations:** 1 Department of Molecular Virology, Tokyo Medical and Dental University, Tokyo, Japan; 2 Division of Ultrastructural Virology, International Research Center for Infectious Diseases, Institute of Medical Science, The University of Tokyo, Tokyo, Japan; 3 PRESTO, Japan Science and Technology Agency, Saitama, Japan; 4 Laboratory of Ultrastructural Virology, Institute for Frontier Life and Medical Sciences, Kyoto University, Kyoto, Japan; 5 Discovery Research Laboratory for Core Therapeutic Areas, Shionogi Pharmaceutical Research Center, Shionogi & CO., LTD, Osaka, Japan; 6 Department of Immunology, National Institute of Infectious Diseases, Tokyo, Japan; 7 AIDS Research Center, National Institute of Infectious Diseases, Tokyo, Japan; 8 Department of Medical Technology, School of Human Sciences, Tokyo University of Technology, Tokyo, Japan; Fred Hutchinson Cancer Research Center, UNITED STATES

## Abstract

Regulation of capsid disassembly is crucial for efficient HIV-1 cDNA synthesis after entry, yet host factors involved in this process remain largely unknown. Here, we employ genetic screening of human T-cells to identify maternal embryonic leucine zipper kinase (MELK) as a host factor required for optimal uncoating of the HIV-1 core to promote viral cDNA synthesis. Depletion of MELK inhibited HIV-1 cDNA synthesis with a concomitant delay of capsid disassembly. MELK phosphorylated Ser-149 of the capsid in the multimerized HIV-1 core, and a mutant virus carrying a phosphorylation-mimetic amino-acid substitution of Ser-149 underwent premature capsid disassembly and earlier HIV-1 cDNA synthesis, and eventually failed to enter the nucleus. Moreover, a small-molecule MELK inhibitor reduced the efficiency of HIV-1 replication in peripheral blood mononuclear cells in a dose-dependent manner. These results reveal a previously unrecognized mechanism of HIV-1 capsid disassembly and implicate MELK as a potential target for anti-HIV therapy.

## Author summary

Phosphorylation of the HIV-1 capsid has long been known to regulate viral uncoating and cDNA synthesis processes, but the cellular kinases responsible for this have remained unidentified. Here, we report that a host cell kinase MELK dictates optimal capsid disassembly through phosphorylation of Ser-149 in the multimerized HIV-1 core, which leads to efficient viral cDNA synthesis in target cells. The phosphorylation-mimetic capsid mutation of Ser-149 caused aberrant capsid disassembly and too-early completion of reverse transcription, and impeded nuclear entry of HIV-1 cDNA, suggesting the importance of well-ordered capsid disassembly in the early stages of viral replication. This discovery will facilitate understanding of the functional link among virus uncoating, reverse transcription and nuclear entry, and is expected to contribute to developing a novel strategy for AIDS therapy.

## Introduction

During the course of human immunodeficiency virus type 1 (HIV-1) infection, the virus encounters numerous bottlenecks constituted by a variety of host cell proteins essential for or inhibitory to HIV-1 replication [1]. HIV-1 particles must attach to and fuse with the plasma membrane of target cells, releasing the viral core into the cytoplasm. Shortly after entry, the HIV-1 capsid (CA), a major component of the viral core, starts dissociating from the core [reviewed by [2–5]]. It has been shown that optimal dissociation of CA from the HIV-1 core is required for efficient viral cDNA synthesis in target cells [6–8]. Thus, (i) Rhesus monkey TRIM5α abrogates productive reverse transcription (RT) by accelerating the disassembly of CA [9, 10]; (ii) CA mutations that impair HIV-1 infection are unable to achieve proper uncoating and RT [6–8]; (iii) The prevention of RT with RT inhibitors causes CA disassembly delay [11, 12]; and (iv) Uncoating of the HIV-1 CA core is triggered following first strand transfer of reverse transcription [13]; (v) The progression of reverse transcription causes morphological and mechanical changes in the HIV-1 cores [14]. Overall, these observations suggest that proper dissociation of CA is functionally linked to reverse transcription of HIV-1. This is also supported by studies showing that cytoplasmic accumulation of CPSF6 restricts HIV-1 infection through abnormal stabilization of the HIV-1 core [15–17]. HIV-1 CA is likely to interact with multiple host cell factors during uncoating and trafficking to the nucleus [3–5, 18]. However, it remains poorly understood how the HIV-1 core dissociation process is triggered and regulated by host factors. One important consideration lies in the phosphorylation of CA because previous studies have shown that its phosphorylation plays pivotal roles in the viral life cycle [19–21]. For example, Ser-109 located in the amino-terminal domain, Ser-149 in the flexible linker and Ser-178 in the carboxy-terminal domain have been identified as major phosphoacceptor sites in CA which are essential for virus replication [19]. Alanine substitution at Ser-109, Ser-149 or Ser-178 reduces the phosphorylation level of CA in cell-free virions and inhibits efficient viral cDNA synthesis [19, 22]. Furthermore, these mutations cause aberrant CA assembly or impaired core stability [21–23]. Phosphorylation of other amino acid residues in CA has also been reported to contribute to viral replication [24–27]. In terms of capsid phosphorylation by virion-associated kinases, the catalytic subunit of cAMP-dependent protein kinase (C-PKA) was reported to interact with and phosphorylate CA, and thus regulate viral infectivity, although the residues that were phosphorylated were not identified [28, 29]. A recent study showed that virion-associated extracellular signal-regulated kinase 2 (ERK2) phosphorylates Ser-16 in CA [30], while an earlier study showed that HIV-1 CA is not a direct substrate of MAPK/ERK2 [19]. Thus, the significance of the phosphorylation of each of the amino acid residues in CA and the contribution of host cell kinases to HIV-1 replication remains to be fully elucidated. In the current study, we performed a genome-wide RNAi screen in a human T-cell line to identify host factors that contribute to HIV-1 infection. We found that a cellular kinase, MELK, is responsible for phosphorylation of the HIV-1 CA in target cells.

## Results

### Genome-wide RNAi screen identifies MELK as a host factor required for HIV-1 replication

**Fig 1:**
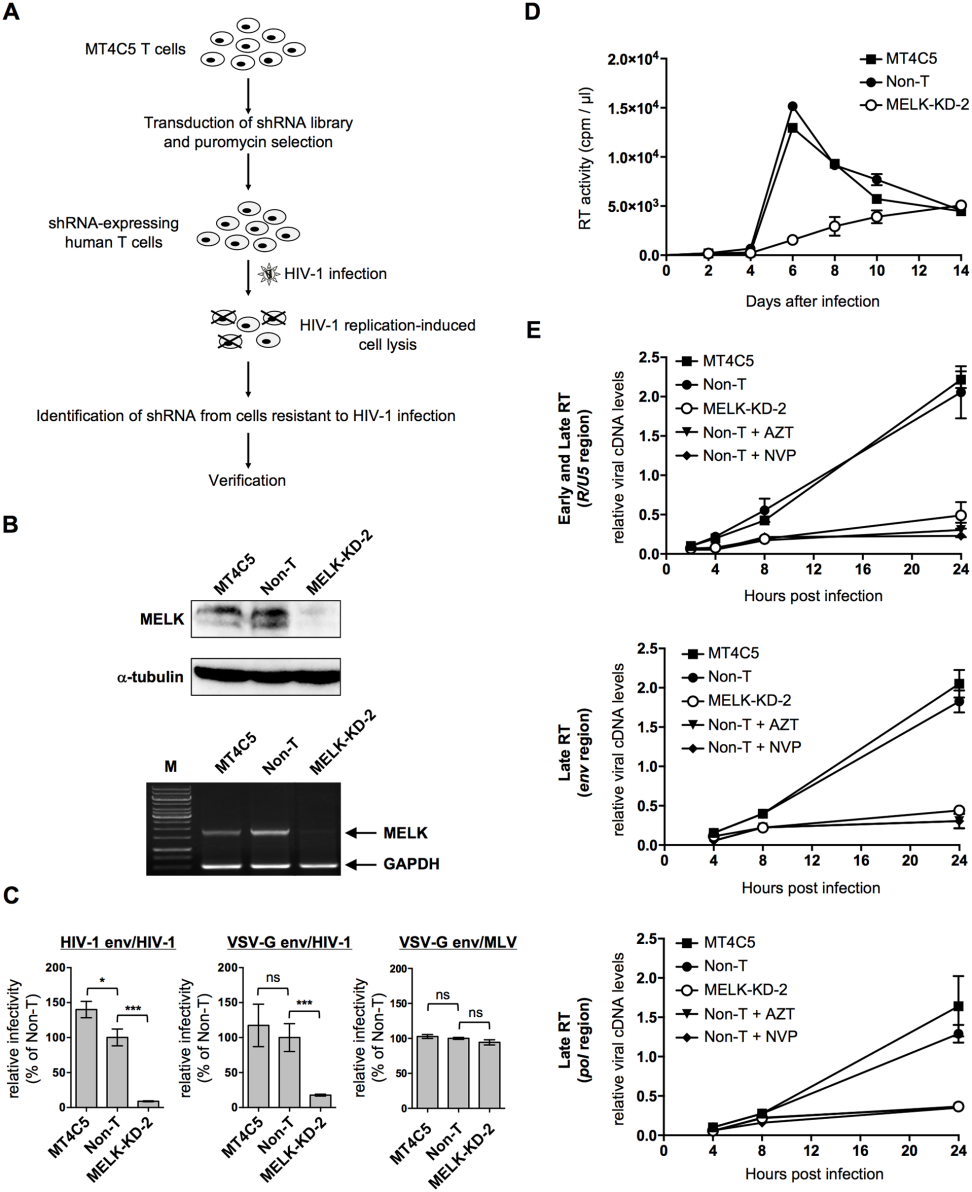
Identification of MELK as an essential host factor for HIV-1 infection of human cells. **(A)** Schematic summary of the genome-wide RNAi screen. **(B)** Immunoblot analysis monitoring MELK expression in MT4C5 cells stably expressing either non-target (Non-T) or MELK-specific shRNA (MELK-KD-2) (upper panel). Semi-quantitative RT-PCR analysis of *MELK* mRNA expression (lower panel). A primer set for amplification of *GAPDH* mRNA was included in each reaction as an internal control (GAPDH). **(C)** MT4C5, Non-T and MELK-KD-2 cells were infected with HIV-1-env- or VSV-G-env-pseudotyped NL4-3luc, or VSV-G-env-pseudotyped MLV-luc. Relative luciferase activities are shown as ratios (%) of the RLU of non-target shRNA MT4C5 cells with standard deviations calculated from five independent experiments. **(D)** Effect of MELK depletion on HIV-1 replication in MT4C5 cells. The virion-associated RT activity was monitored at the indicated time points in culture supernatants of MT4C5 (closed squares), Non-T (closed circles) and MELK-KD-2 (open circles) cells. **(E)** Quantitative DNA-PCR analyses of viral cDNA metabolism after HIV-1 infection of MT4C5-derived cells in the presence or absence of AZT (5 μM) or NVP (10 μM). Total DNA was extracted from a portion of the cells 2 h after infection and early viral cDNA synthesis was quantified by real-time PCR with a primer set recognizing the *R/U5* region (top panel). Total DNA was extracted at the indicated time points (4, 8 and 24 h) and analyzed for the amount of late viral cDNA with a primer set recognizing the *R/U5* region (top panel), *env* (middle panel) or *pol* (bottom panel) regions. The ratios of each viral cDNA level to beta-globin DNA level are given. Experiments were performed at least three times and error bars are standard deviations calculated from three independent experiments. Statistical significance was determined by one-way analysis of variance (ANOVA) with Dunnett’s multiple comparison test **(C)**. ns, not significant (*P*>0.05); *P<0.05, **P<0.01, ***P<0.001.

To identify host cell factors involved in HIV-1 replication in human cells, we employed a genome-wide RNAi screen in the MT4C5 lymphoid cell line, a derivative of MT4 cells expressing CCR5 and susceptible to infection with CXCR4-tropic and CCR5-tropic HIV-1 strains. MT4C5 cells were transduced with ten independent pools of puromycin-marked lentivirus vectors expressing a human short hairpin RNA (shRNA) library comprising **>**75,000 shRNAs directed against **>**15,000 human genes in total. Transduced cells were then infected with the HIV-1_NL4-3_ strain which normally kills infected MT4C5 cells. Surviving cells were then studied further (Fig 1A). Using this approach, 32 individual shRNA sequences were obtained that potentially target host factors, including Cyclophilin A and Transportin-SR2 (TNPO3), which are known to be essential host factors [1, 31] (S1 Table). Of these, we have characterized maternal embryonic leucine-zipper kinase (MELK) in detail. This was identified in a sub-pool resistant to HIV-1 infection. MELK is a member of the AMP-activated protein kinase-related Ser/Thr protein kinase family [32]. Previous reports indicated that MELK is expressed mainly in the cytoplasm and is involved in different cellular processes such as cell-cycle progression, cell proliferation and pre-mRNA splicing [33–37]. However, involvement of MELK in HIV-1 replication has not been reported.

To determine whether endogenous MELK is involved in HIV-1 infection, we depleted this enzyme from MT4C5 cells (MELK-KD). Lentivirus-mediated stable expression of a MELK-targeting shRNA, but not that of non-targeting shRNA (Non-T), suppressed expression of *MELK* mRNA (Fig 1B, lower panel, compare Non-T and MELK-KD-2) and protein (Fig 1B, upper panel, compare Non-T and MELK-KD-2). Parental MT4C5 and MELK-KD cells showed no significant difference in growth and surface expression of CD4 and CXCR4 (S1 Fig). Analyses of cell-cycle progression using propidium iodide DNA staining revealed no significant differences between Non-T and MELK-KD cells after release from synchronization by demecolcine, which arrests cells in mid-metaphase of the cell cycle (S2 Fig). Depletion of MELK markedly reduced the infectivity of HIV-1- or VSV-G- envelope-pseudotyped NL4-3luc, although there was a small difference in the HIV-1 env/HIV-1 infectivity between parental and Non-T cells. The results suggest that MELK is required for HIV-1 infection during a post-entry stage (Fig 1C, left and middle panels). Inhibition of HIV-1 infection by MELK depletion with three different MELK-targeting shRNAs, 293-MELK-KD-1, 293-MELK-KD-2 and 293-MELK-KD-3, was dependent on its level of expression in the single-round infection of HEK293 cells with VSV-G pseudotyped HIV-1, reducing the likelihood of an off-target effect of the shRNA (S3A–S3C Fig). Importantly, similar results were obtained with MELK-depleted and CD3/CD28-stimulated peripheral blood lymphocytes (PBL) (S3D–S3F Fig). In contrast, MELK depletion did not affect the infectivity of the VSV-G-pseudotyped murine leukemia virus (MLV)-based vector (Fig 1C, right panel). HIV-1 replication in MT4C5 cells with the replication-competent NL4-3 virus was markedly inhibited by MELK depletion (Fig 1D). Viral DNA synthesis by replication-competent HIV-1 proceeded more slowly than by VSVG-pseudotyped HIV-1, continuing until 24 h after infection, as previously reported [38, 39]. Of note, MELK depletion did not significantly affect the amount of immediate early viral cDNA quantified at the *R/U5* region 2 h post-infection (Fig 1E, upper panel), but profoundly reduced it thereafter by approximately 80% compared to Non-T (Fig 1E, upper panel). This is because amplification of the *R/U5* region includes both Early and Late RT products. Similar results were obtained by quantifying viral cDNA at the *pol* and *env* regions as Late RT products (approximately 80% reduction compared to Non-T) (Fig 1E, middle and lower panels). Viral cDNA synthesis after HIV-1 infection in the presence of the HIV-1 reverse transcriptase inhibitors azidothymidine (AZT) or nevirapine (NVP) was markedly reduced (Fig 1E, compare Non-T, Non-T + AZT and Non-T + NVP). Collectively, these results indicate that MELK is a host factor required for efficient viral cDNA synthesis.

### MELK depletion delays HIV-1 capsid disassembly

**Fig 2:**
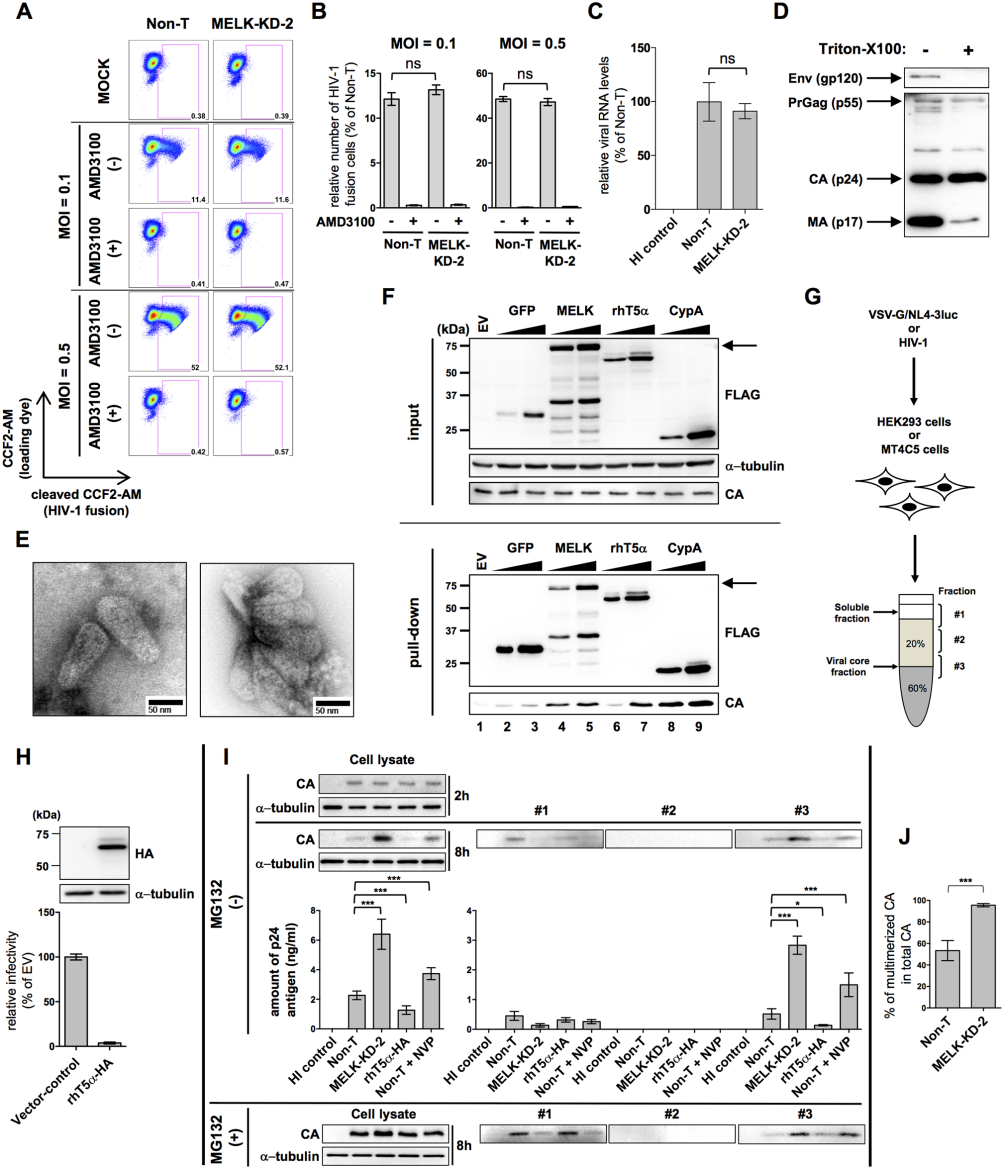
Depletion of MELK delays HIV-1 CA disassembly. **(A)** Non-T or MELK-KD-2 MT4C5 cells were mock-infected or infected with 100 or 500 ng of p24-measured amounts of NL4-3 virions containing BlaM-Vpr, based on the measured amount of p24, in the presence or absence of AMD3100 (100 nM). They were then analyzed in the fusion assay by flow cytometry using a violet laser to excite CCF2. Each experiment was performed in triplicate, repeated three times and one set of representative data is shown. **(B)** Relative numbers of BlaM^+^ MELK-KD-2 MT4C5 cells are shown as percentages (%) of Non-T MT4C5 cells with standard deviations calculated from three independent experiments. **(C)** Virion-associated viral RNA was quantified by quantitative RT-PCR 2 h after infection of Non-T or MELK-KD-2 MT4C5 cells with wild-type HIV-1. Error bars indicate the standard deviations calculated from five independent experiments. (**D)** Immunoblot analysis of envelope-stripped HIV-1 cores. Concentrated virions were subjected to step-gradient centrifugation in the absence (-) or presence (+) of 0.1% of Triton-X100. **(E)** Electron micrographs showing envelope-stripped cores of HIV-1. TEM images of a negatively stained envelope-stripped core of HIV-1 prepared from HIV-1_NL4-3_ virions. Bars indicate 50 nm. **(F)** Immunoblot analyses showing MELK bound to envelope-stripped cores of HIV-1. HeLa cells were transfected with pCAG-OSF (lane 1) or increasing amounts of pCAG-OSF-GFP (lanes 2 to 3), pCAG-OSF-MELK (lanes 4 to 5), pCAG-FOS2-rhT5α (lanes 6 to 7) or pCAG-OSF-CypA (lanes 8 to 9). Purified OSF- or FOS2-tagged proteins were incubated with envelope-stripped cores and complex formation was assessed. Masses of molecular weight standards are indicated on the left. Arrows indicate the position of MELK in the gel. **(G)** Schematic diagram of the fate-of-capsid assay. **(H)** Forced expression of rhesus Trim5α (rhT5α) inhibits HIV-1 replication in human T cells. Cell lysates were prepared from MT4C5 cells transduced with empty lentivirus (vector-control) or lentivirus for C-terminally HA-tagged rhesus Trim5α expression (rhT5α-HA) and processed for immunoblotting with anti-HA (HA) and anti-alpha-tubulin (α-tubulin) antibodies. Experiments were performed at least three times and one representative set of data is shown (upper panels). Vector-control and rhT5α-HA cells were infected with VSV-G-env-pseudotyped NL4-3luc. Relative luciferase activity is shown as a percentage (%) of the RLU of vector-control cells with standard deviations calculated from five independent experiments (lower panel). **(I)** Effect of MELK depletion on the fate of the HIV-1 CA in MT4C5 cells. Non-T, MELK-KD-2, MT4C5 cells expressing rhT5α (rhT5α), or Non-T cells treated with 10 μM nevirapine (Non-T + NVP) were infected with wild-type HIV-1 for 8 h in the presence or absence of 10 μM MG132 (MG132 [+] or MG132 [–]). HIV-1 stock inactivated by incubation at 65°C for 30 min was used as a negative control (HI control). Cell lysates were subjected to 20%–60% step-gradient centrifugation and three fractions were collected from the top (fraction #1), middle (fraction #2) and interface between the 20% and 60% sucrose layers (fraction #3). Aliquots of each fraction were processed for immunoblotting with anti-p24 antibody (CA) (upper panel). Experiments were performed at least five times and one representative set of data is shown. The amount of CA in each fraction in the absence of MG132 was quantified by HIV-1 p24 ELISA (lower panel). Error bars indicate the standard deviations calculated from five independent experiments. **(J)** Percentage of the pelletable CA (fraction #3) within total CA in the absence of MG132 was calculated based on the p24 ELISA data shown in Fig 2H. Total CA denotes the sum of the amount of p24 antigen which was calculated based on the p24 ELISA data of fractions #1, #2, and #3. Error bars represent the standard deviations calculated from five independent experiments. Statistical significance was determined by unpaired two-tailed Student’s *t* test **(B**, **C**, **H** and **J)**, or one-way analysis of variance (ANOVA) with Dunnett’s multiple comparison test **(I)**. ns, not significant (*P*>0.05); *P<0.05, **P<0.01, ***P<0.001.

We first investigated whether MELK affects HIV-1 entry using the HIV-1 virion fusion assay with β-lactamase-Vpr chimeric protein incorporated into HIV-1 virions. This approach revealed similar efficiencies of HIV-1 entry into control Non-T and MELK-KD MT4C5 cells and a marked inhibition of HIV-1 fusion in the presence of the CXCR4 antagonist AMD3100 [40] (Fig 2A and 2B, compare Non-T and MELK-KD-2). Quantitative RT-PCR assays also showed similar amounts of incoming viral genome RNA 2 h post infection (Fig 2C, compare Non-T and MELK-KD-2). We next assessed whether it is involved in proper disassembly of the viral CA. To determine whether the viral core interacts with MELK, we purified One-STrEP-FLAG-(OSF)-tagged MELK protein expressed in HeLa cells, and viral cores from cell-free virions. The HIV-1 envelope was removed from virions and envelope-stripped cores were enriched by ultracentrifugation through a discontinuous 10% and 30% sucrose gradient with 0.1% Triton X-100 in the 10% sucrose layer, as previously reported [20] (Fig 2D). The envelope-stripped cores were characterized by transmission electron microscopy (TEM) showing recognizable ~100 nm cone-shaped structures similar to authentic HIV-1 cores (Fig 2E). Purified OSF (N-terminal)-tagged Cyclophilin A (CypA) or FLAG-One-STrEP (FOS) (C-terminal)-tagged rhesus monkey Trim5α (rhT5α) proteins, known to be HIV-1 core-binding proteins [9, 41–43], were used as positive controls for binding to the HIV-1 core. Pull-down assays revealed that OSF-tagged CypA or FOS2-tagged rhT5α, but not OSF-tagged Green Fluorescent Protein (GFP), significantly interacted with the envelope-stripped core in a dose-dependent manner (Fig 2F, lower panel CA, compare lanes 2–3, 6–7 and 8–9). Similar to CypA and rhT5α, MELK interacted with the envelope-stripped core (Fig 2F, lower panel CA, compare lanes 2–3 and 4–5). Immunoblot analyses revealed that the envelope-stripped cores, but not enveloped virions or the CA monomer, interacted in vitro with OSF-tagged MELK (S4 Fig). We next tested whether MELK affects the stability of the HIV-1 core after viral entry, using a fate-of-capsid assay, as described previously [39] and summarized in Fig 2G. Previous reports showed that uncoating was closely linked to reverse transcription, using VSVG-pseudotyped lentivirus vectors that enter target cells in large numbers [13, 18, 44–46] and far more quickly by endocytosis than the replication-competent HIV-1 does through CD4- and CXCR4-mediated fusion with the plasma membrane. As far as we know, we used for the first time replication-competent HIV-1 in the fate of capsid assay to demonstrate how MELK acts on HIV-1 in an experimental setting more relevant to human pathology. We chose to focus on the time point 8 h post infection for this fate-of-capsid assay for the following reasons: (i) previous reports showed that reverse transcription products reached a maximum 24 h after infection with replication-competent HIV-1 [38, 39]; (ii) our requirement studies indicated that the amount of total intracellular CA 4 h after infection remained so small that it was impossible to detect pelletable CA by western blotting following ultracentrifugation (S5 Fig). Previous studies had shown that ectopic expression of the rhT5α protein accelerated uncoating and restricted HIV-1 infection, and that a reverse transcriptase inhibitor, NVP, delayed CA disassembly [9, 11, 12, 47]. To confirm the validity of this fate-of-capsid assay, we established MT4C5 cell pools stably expressing C-terminally hemagglutinin (HA)-tagged rhT5α (rhT5α-HA) (Fig 2H, upper panel). As reported previously [47], rhT5α strongly inhibited HIV-1 infection (Fig 2H, lower panel). Control Non-T, MELK-KD, rhT5α-HA-expressing cells and Non-T cells treated with NVP were infected with wild-type HIV-1. We found that the amount of HIV-1 cores in HIV-1-infected cells expressing rhT5α-HA 8 h post-infection was significantly lower than in control cells (Non-T) (Fig 2I, panel CA of fraction #3, MG132[–], compare Non-T and rhT5α-HA). We also found that treatment of Non-T cells with NVP 8 h post-infection caused a marked delay of CA disassembly (Fig 2I, panel CA of fraction #3, MG132[–], compare Non-T and Non-T + NVP). Immunoblot analyses 2 h post-infection revealed that the levels of CA protein in the cell lysates from Non-T or MELK-KD cells were similar, indicating similar efficiency of HIV-1 entry (Fig 2I, panel CA of cell lysate 2 h, MG132[–], compare Non-T and MELK-KD-2). However, immunoblotting 8 h post-infection revealed that the amount of HIV-1 CA in the cell lysate from MELK-KD cells was significantly larger than that from Non-T cells in the absence of MG132 (Fig 2I, panel CA of cell lysate 8 h, MG132[–], compare Non-T and MELK-KD-2). This difference was confirmed in a quantitative manner by p24 ELISA (Fig 2I, left lower bar graph, MG132 [–], compare Non-T and MELK-KD-2). This suggests that CA monomers dissociated from multimerized cores undergo degradation in living cells. Consistent with a previous report [48], degradation of incoming CA protein in our hypotonic lysis buffer was accelerated by rhT5α (Fig 2I, panel CA of cell lysate 8h, MG132[–], compare Non-T and rhT5α-HA). The report also showed that the proteasome inhibitors MG132 and lactacystin caused markedly increased steady-state levels of incoming CA protein in the cytosol of HeLa cells expressing non-restrictive human Trim5α or rhT5α [48]. We therefore used the proteasome inhibitor MG132 to retain the CA in infected cells as far as possible in order to show how much CA was actually present in infected cells at the time of the assay. Indeed, the inhibition of CA degradation by the proteasome revealed that similar amounts of CA were present in infected cells (Fig 2I, panel CA of cell lysate 8 h, MG132[+], compare Non-T, MELK-KD-2, rhT5α-HA and Non-T + NVP). Importantly, more viral cores were found 8 h post-infection in MELK-KD cells than in Non-T cells regardless of proteasome inhibition, indicating that depletion of MELK in MT4C5 cells impaired the dissociation of CA from the HIV-1 core (Fig 2I, panel CA of fraction #3, MG132 [–] and MG132[+], compare Non-T and MELK-KD-2). Accordingly, the ratio of pelletable CA of HIV-1 cores to total CA in MELK-KD cells quantified by p24 ELISA in the absence of MG132 was significantly increased compared to Non-T cells (Fig 2J). To determine whether the effects of MELK on CA disassembly were cell type-specific, similar experiments were performed with the cells used in S6A–S6C Fig. Immunoblotting revealed that CA in the cell lysate (S6B Fig, left upper panel) or derived from HIV-1 cores (S6B Fig, right upper panel, fraction #3) was significantly increased in HEK293 cells 8 h after VSV-G-pseudotyped HIV-1 infection. This was accomplished without any significant difference in the efficiency of VSV-G-pseudotyped HIV-1 entry, as in MT4C5 cells (S6A Fig, compare Non-T and 293-MELK-KD-3). Again, confirmation was obtained by quantitative p24 ELISA (S6B Fig, bottom panels). As in MT4C5 cells, the proportion of pelletable CA in 293-MELK-KD-3 cells was significantly greater than in control Non-T cells (S6C Fig). Proteasome inhibition by MG132 treatment indicated that there were similar amounts of CA in infected cells, suggesting that MELK depletion stabilizes intracellular CA protein as in MT4C5 cells (S6D Fig). CA derived from viral cores in MELK-KD MT4C5 and MELK-KD HEK293 cells were increased relative to control cells at all time points examined (S6E Fig). These results clearly indicate that MELK is required for optimal HIV-1 capsid disassembly in newly infected cells.

### Catalytic activity of MELK regulates HIV-1 replication

**Fig 3:**
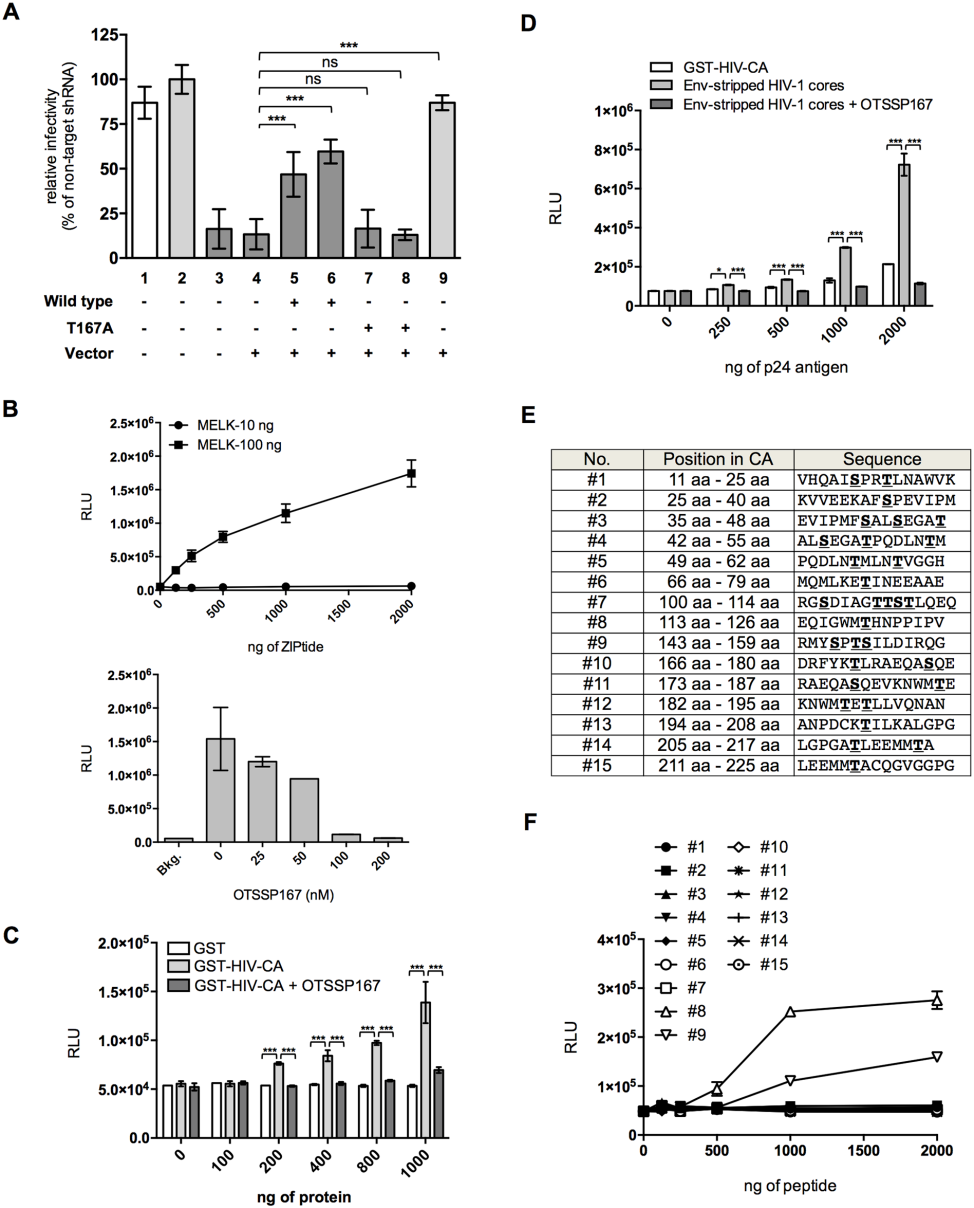
Catalytic activity of MELK is required for HIV-1 infection. **(A)** Effect of exogenous wt or T167A MELK on single-round HIV-1 infection. VSV-G-pseudotyped NL4-3luc was used to infect parental MT4C5 (white bar: lane 1), Non-T (gray bars: lanes 2 and 9) and MELK-KD-1 (dark gray bars: lanes 3 to 8) cells transduced with control vector (lane 4), wild**-**type MELK (lanes 5 and 6) or catalytically inactive T167A MELK mutant (lanes 7 and 8) (see also S7 Fig). Two independent MELK-KD-1 cell pools expressing wild**-**type MELK (lanes 5 and 6) or T167A MELK mutant (lanes 7 and 8) were used. Error bars indicate the standard deviations calculated from five independent experiments. **(B)** *In vitro* luminescent kinase assay with recombinant active MELK (10 or 100 ng) and increasing amounts of ZIPtide, a substrate for MELK (upper panel). Phosphorylation of the substrate was monitored as the amount of ADP produced during the kinase reaction. Effect of OTSSP167, a MELK kinase inhibitor, on *in vitro* MELK kinase activity (lower panel). Error bars indicate the standard deviations calculated from three independent experiments. **(C)** *In vitro* luminescent kinase assay with recombinant active MELK and increasing amounts of the indicated GST fusion proteins in the presence or absence of OTSSP167 (100 nM). Mean values from five independent experiments are shown. Error bars indicate the standard deviations calculated from five independent experiments. **(D)** *In vitro* luminescent kinase assay with recombinant active MELK and increasing amounts of the indicated substrates in the presence or absence of OTSSP167 (100 nM). Phosphorylation of proteins was monitored as in **(C)**. Error bars indicate the standard deviations calculated from five independent experiments. **(E)** List of fifteen different peptides containing serine or threonine residues in HIV-1 CA. **(F)** *In vitro* luminescent kinase assay with recombinant active MELK and increasing amounts of each peptide shown in **(E)**. Phosphorylation of the peptides was monitored as in **(B)**. Experiments were performed at least three times and error bars are standard deviations calculated from three independent experiments. Statistical significance was determined by one-way analysis of variance (ANOVA) with Dunnett’s multiple comparison test **(A)**, or two-way ANOVA with Tukey’s multiple comparison test **(C** and **D)**. ns, not significant (*P*>0.05); *P<0.05, **P<0.01, ***P<0.001.

Transduction of MELK-depleted MT4C5 expressing an shRNA targeting the 3′-untranslated region (3′-UTR) of MELK (MT4C5-MELK-KD-1) with a lentivirus vector capable of expressing wild-type MELK substantially restored HIV-1 infectivity in two independent cell pools (Fig 3A, compare lanes 4 and 5 or 6). In contrast, a MELK mutant (T167A) that lacks catalytic activity [37] failed to do so (Fig 3A, compare lanes 4 and 7 or 8), suggesting that the kinase activity of MELK is required for efficient HIV-1 replication. Depletion of endogenous MELK as well as forced expression of exogenous MELK was verified by RT-PCR and immunoblotting (S7 Fig). To determine whether CA is a substrate for MELK, we prepared recombinant CA fused to GST (GST-HIV-CA) and employed an *in vitro* luminescent kinase assay, in which the amount of ADP produced in the kinase reaction was quantified (for details, see “Materials and methods”). MELK phosphorylated ZIPtide, a substrate for MELK [49] (Fig 3B, upper panel), which was inhibited by the small-molecule MELK inhibitor OTSSP167 [50] in a dose-dependent manner (Fig 3B, lower panel). As shown in Fig 3C, MELK significantly phosphorylated GST-HIV-CA, but not the control GST protein (compare GST and GST-HIV-CA) or GST-HIV-CA in the presence of OTSSP167 (compare GST-HIV-CA and GST-HIV-CA + OTSSP167). We obtained similar results with the GST-free recombinant HIV-1 CA protein (S8 Fig). These results suggest that CA is a substrate for MELK. Because the results shown in Fig 2F implied that MELK preferentially recognized multimerized CA cores, we next determined whether MELK phosphorylates CA in a structure-dependent manner. *In vitro* luminescent kinase assays revealed that env-stripped HIV-1 cores were much more efficiently phosphorylated by MELK than GST-HIV-CA (Fig 3D, compare GST-HIV-CA and Env-stripped HIV-1 cores). To explore which Ser or Thr residue(s) in CA can be phosphorylated by MELK, we generated fifteen peptides that covered all the regions containing Ser or Thr residues in CA (Fig 3E). *In vitro* luminescent kinase assays revealed that peptides #8 and #9 were phosphorylated by MELK in a substrate dose-dependent manner (Fig 3F). These results suggest that Thr-119, Ser-146, Thr-148 and Ser-149 of CA could be phosphorylation targets of MELK.

### Phosphorylation of Ser-149 in CA by MELK regulates HIV-1 uncoating in target cells

**Fig 4:**
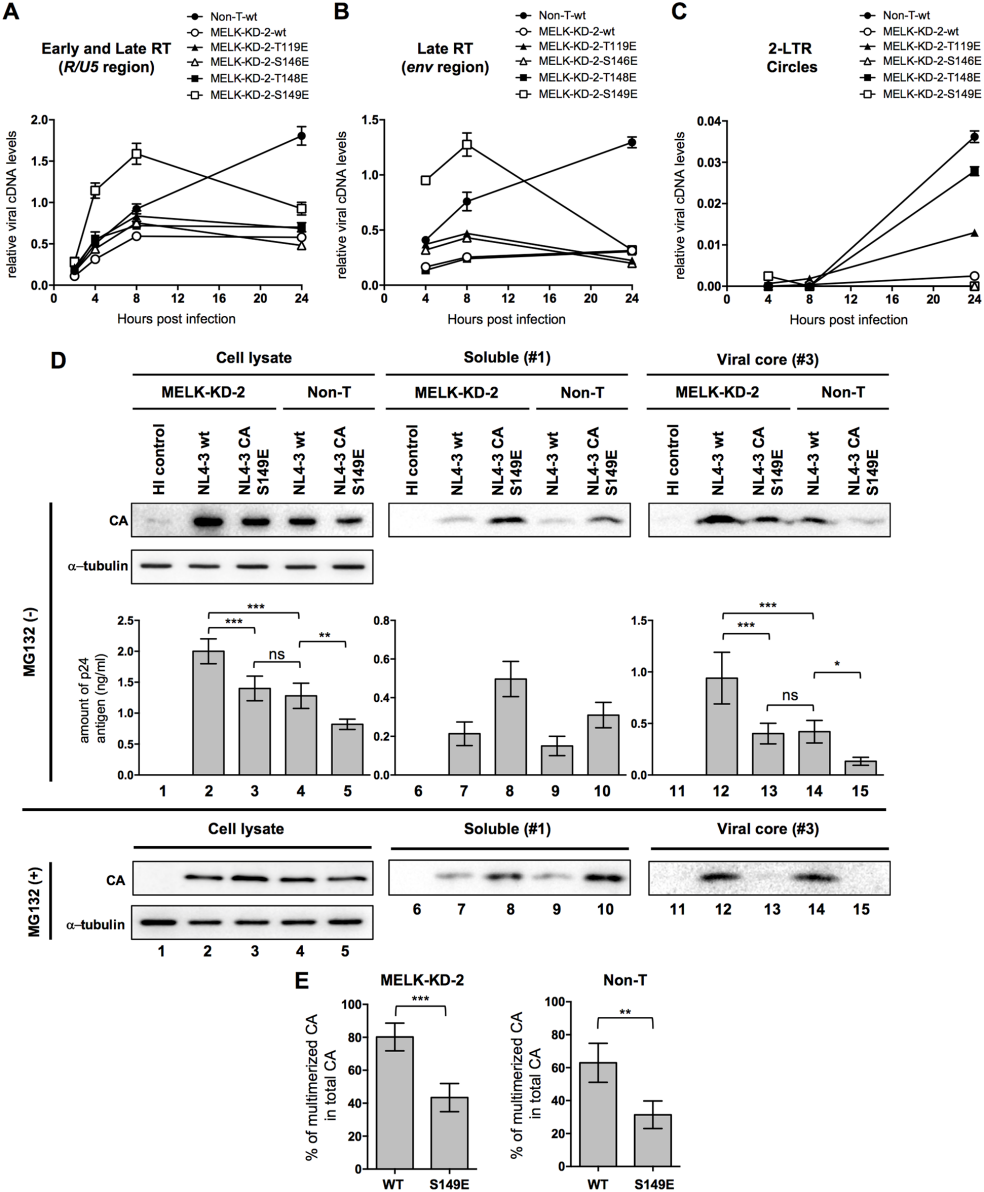
Phosphorylation of Ser-149 in CA regulates CA disassembly and viral cDNA synthesis in human T cells. **(A-C)** Quantitative DNA-PCR analyses of viral cDNA metabolism after HIV-1 infection of MT4C5 cells. Total DNA was extracted from non-target shRNA (Non-T) or MELK-depleted (MELK-KD-2) MT4C5 cells at the indicated time points (2, 4, 8 and 24 h) after infection with wild-type or the indicated mutants of HIV-1 and analyzed for the amounts of the *R/U5* region as early viral cDNA **(A)**, the *env* region as late viral cDNA **(B)** and the 2-LTR circle form **(C)**. Experiments were performed at least three times and error bars are standard deviations calculated from three independent experiments. The ratios of each viral cDNA level to beta-globin DNA level are given. **(D)** Fate-of-capsid assays with non-target shRNA (Non-T) or MELK-KD-2 MT4C5 cells infected with NL4-3 or its S149E CA mutant for 8 h in the presence or absence of 10 μM MG132 (MG132 [+] or MG132 [–]). HIV-1 stock inactivated by incubation at 65°C for 30 min was used as a negative control (HI control). Cell lysates were prepared and analyzed as in Fig 2I. Aliquots of input, fraction #1 and #3 were processed for immunoblotting with anti-p24 antibody (CA). The amount of CA in each fraction in the absence of MG132 was quantified by HIV-1 p24 ELISA (MG132 [–], lower panels). Experiments were performed five times and one representative set of data is shown. **(E)** Percentages of pelletable CA (fraction #3) within total CA in the absence of MG132 were calculated based on the p24 ELISA data shown in **Fig 4D**. Total CA denotes the sum of the amount of p24 antigen which was calculated based on the p24 ELISA data of fractions #1, #2, and #3. Error bars represent the standard deviations calculated from five independent experiments. Statistical significance was determined by one-way analysis of variance (ANOVA) with Tukey’s multiple comparison test **(D)**, or unpaired two-tailed Student’s *t* test **(E)**. ns, not significant (*P*>0.05); *P<0.05, **P<0.01, ***P<0.001.

We next explored whether an amino-acid substitution that mimics phosphorylation of each serine or threonine residue, Thr-119, Ser-146, Thr-148 and Ser-149, counteracts the delay of CA disassembly and reduction in viral cDNA synthesis caused by MELK depletion. We generated four mutant pNL4-3 proviral molecular clones in which each Ser or Thr residue was substituted by a glutamic acid residue so that each mutation mimics constitutive phosphorylation of the site. Mutant HIV-1 bearing T119E, S146E, T148E, or S149E mutations in CA were used to evaluate the efficiency of viral cDNA synthesis in MELK-KD MT4C5 cells. The amount of each input CA-mutated virus was normalized by its RT activity. HIV-1 bearing T119E, S146E or T148E poorly restored early (Fig 4A) and late (Fig 4B) cDNA synthesis in MELK-KD MT4C5 cells. In contrast, viral cDNA synthesis after infection with HIV-1 bearing the S149E mutation was robustly restored, and even at an earlier time point than wild-type HIV-1 (approximately 1.7-fold increase compared to Non-T-wt, 8 h post-infection) (Fig 4A and 4B, MELK-KD-2-S149E). The S149E mutation did not significantly alter the amount of incoming HIV-1 RNA in MELK-KD or control Non-T MT4C5 cells (S9G Fig, compare NL4-3wt and NL4-3 CA S149E). In control MT4C5 cells expressing non-target shRNA, only the S149E mutation caused an earlier peak and subsequent downturn in viral cDNA synthesis similar to that in MELK-KD MT4C5 cells (S9A–S9D Fig). This suggests that phosphorylation of Ser-149 is likely to play an important role in the initiation of viral cDNA synthesis. Despite maintenance of efficient cDNA synthesis by the S149E mutant, production of the 2-LTR circular form of viral cDNA, a marker for nuclear import, was undetectable (Fig 4C, MELK-KD-2-S149E). This shows that this CA mutation promotes viral cDNA synthesis, but does not favor nuclear import. HIV-1 bearing T119E or T148E but not S146E mutations appeared to partially restore production of the 2-LTR circular form (Fig 4C), suggesting that although these mutants failed to substantially restore viral DNA synthesis, they were still competent for nuclear entry. Single-round infection assays using TZM-bl or LuSIV indicator cells revealed very low but detectable infectivity of HIV-1 bearing CA T119E or T148E. However, infection with S146E or S149E CA mutants was undetectable (S10C Fig).

To assess how the S149E mutation in CA influences the kinetics of capsid disassembly, we performed a fate-of-capsid assay in control Non-T and MELK-KD MT4C5 cells. The S149E substitution resulted in a clear decrease of the HIV-1 core not only in MELK-KD cells but also in Non-T cells, indicating that this mutation promoted the CA disassembly irrespective of the presence of MELK (Fig 4D, right panel CA, MG132[–], compare lanes 12–13 and 14–15). Reciprocally, the soluble form of CA was markedly increased in S149E mutant-infected cells relative to NL4-3wt-infected cells (Fig 4D, middle panel CA, MG132[–], compare lanes 7–8 and 9–10). However, this was not due to different efficiencies of HIV-1 entry because the amounts of incoming CA were similar in the presence of MG132, which prevents the degradation of the CA monomer dissociated from the viral core in the cytoplasm (Fig 4D, panel CA of cell lysate, MG132[+], compare lanes 2–3 and 4–5). Consequently, the ratio of pelletable S149E CA to total CA in the absence of MG132 quantified by p24 ELISA was significantly less than that of NL4-3wt in both MELK-KD and control Non-T cells (Fig 4E, compare WT and S149E). Overall, these results suggest that phosphorylation of Ser-149 by MELK is a trigger for CA disassembly in HIV-1 infection.

**Fig 5:**
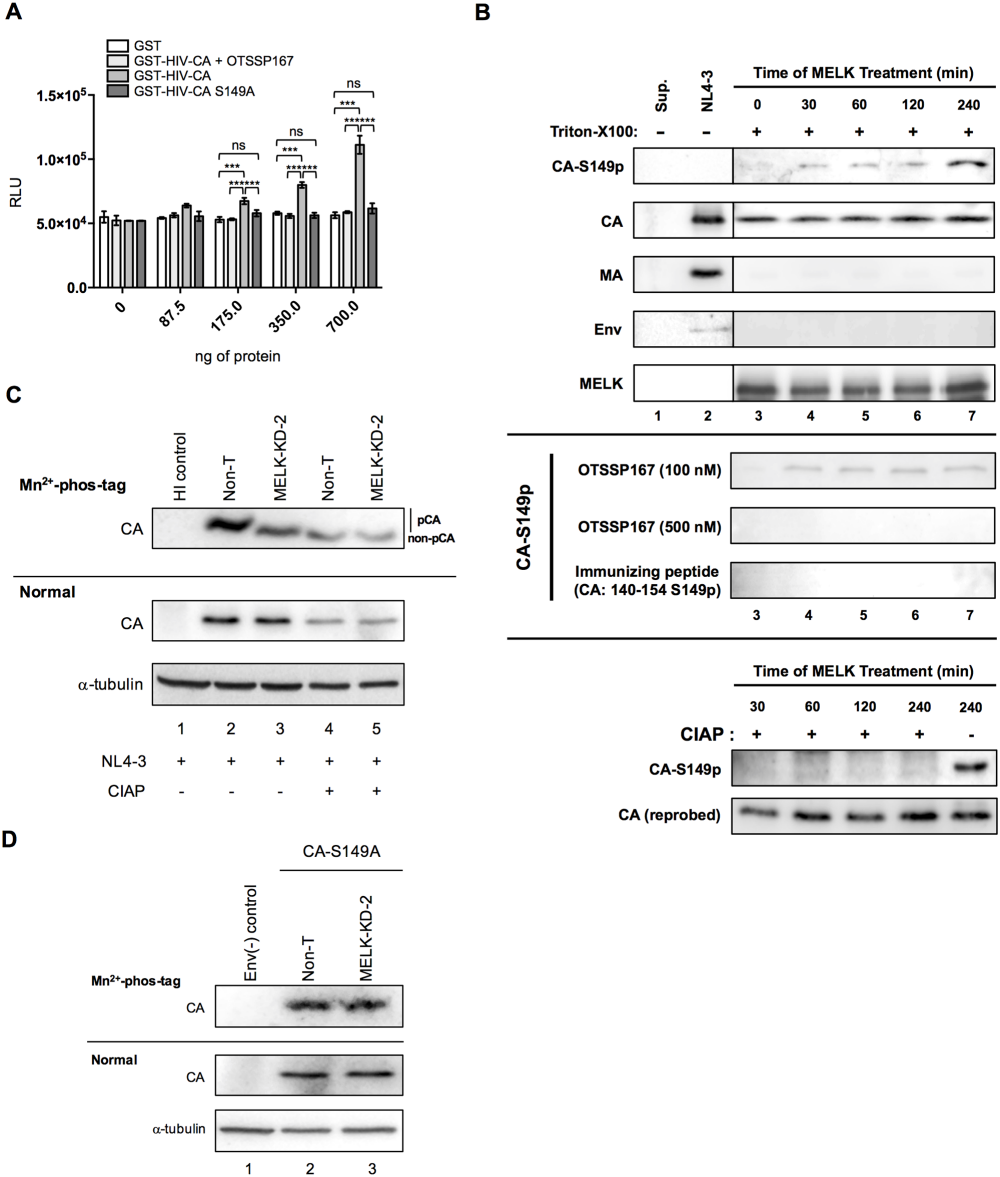
MELK phosphorylates Ser-149 of HIV-1 CA. **(A)** *In vitro* luminescent kinase assay with recombinant active MELK and increasing amounts of the indicated GST fusion proteins in the presence or absence of OTSSP167 (100 nM). Phosphorylation of the proteins was monitored as in Fig 3B. Error bars indicate the standard deviations calculated from five independent experiments. **(B)** Immunoblotting to detect Ser-149 phosphorylation by MELK. Envelope-stripped cores prepared as in Fig 2D were incubated with recombinant MELK for the indicated times. Aliquots of each reaction sample were processed for immunoblotting using anti-phospho-S149-CA (CA-S149p), anti-p24 (CA), anti-p17 (MA), anti-gp120 (Env) or anti-MELK (MELK) (upper 5 panels). Envelope-stripped cores were incubated with recombinant MELK in the presence of 100 nM or 500 nM of OTSSP167 [panel: OTSSP167 (100 nM or 500 nM)]. The samples were also treated with (+) or without (-) 100 U of calf intestine alkaline phosphatase (CIAP) and immunoblotted (lower 2 panels) with CA-S149p (panel: CA-S149p) or with anti-p24 antibody [panel: CA (reprobed)]. Experiments were performed three times and one representative set of data is shown. **(C)** Non-T or MELK-KD-2 MT4C5 cells were infected with VSV-G-pseudotyped HIV-1 or VSV-G-pseudotyped HIV-1 CA-S149A for 8 h. The proteasome inhibitor MG132 (2 μM) was added 5 h after infection to prevent the degradation of CA proteins dissociated from the viral core in the cytoplasm [48]. Cell lysates were separated by SDS-PAGE containing Manganese(II)-Phos-tag (Mn^2+^-phos-tag) or SDS-PAGE without Mn^2+^-phos-tag (Normal), and analyzed by immunoblotting with anti-p24 antibody (CA) or anti-alpha-tubulin antibody (α-tubulin). Cell lysates were incubated for 60 min at 37°C without (lanes 2 and 3) or with (lanes 4 and 5) calf intestine alkaline phosphatase (CIAP). “non-pCA” indicates the position of CA dephosphorylated by CIAP and “pCA” indicates phosphorylated CA. Experiments were performed at least three times and one representative set of data is shown. **(D)** Non-T or MELK-KD-2 MT4C5 cells were infected with VSV-G-pseudotyped HIV-1 CA-S149A for 8 h. Cell lysates were separated as in **(C)** and analyzed by immunoblotting with anti-p24 antibody (CA) or anti-alpha-tubulin antibody (α-tubulin). Similar results were obtained in three independent experiments and a representative result is shown. Statistical significance was determined by two-way analysis of variance (ANOVA) with Tukey’s multiple comparison test **(A)**. ns, not significant (*P*>0.05); *P<0.05, **P<0.01, ***P<0.001.

We also attempted to characterize HIV-1 with an S149A mutation expected to confer refractoriness to phosphorylation by MELK, but the titer of the S149A virus was too low to compare its infectivity with NL4-3wt and S149E mutant. This is consistent with a previous study that the S149A mutation affects the production of infectious virions [23]. We therefore evaluated the infectivity of VSV-G-envelope-pseudotyped NL4-3luc bearing the S149A mutation in CA (VSVG/NL4-3luc CA-S149A) in Non-T and MELK-KD MT4C5 cells. We did this because the infectivity of S149A mutant virus was reported to be rescued specifically by pseudotyping with the VSV envelope protein [22, 23]. The S149A mutation greatly reduced the VSVG/NL4-3luc-derived reporter gene activity in parental and Non-T cells, and also, but less markedly, in MELK-KD cells (S11A Fig, panels MT4C5, Non-T and MELK-KD-2, compare CA-wt and CA-S149A). Additionally, depletion of MELK modestly reduced the reporter gene activity of VSVG/NL4-3luc CA-S149A compared to Non-T control cells (S11B Fig). These findings may in part reflect unidentified dysfunctionality of this mutant CA, but also raise the possibility that MELK regulates HIV-1 replication in a manner in addition to phosphorylating CA Ser-149. A GST-linked S149A CA (GST-HIV-CA S149A) was analyzed using the *in vitro* luminescent kinase assay to determine whether any other residues in CA can be phosphorylated by MELK (Fig 5A). Essentially no phosphorylation could be detected when activated MELK was incubated with increasing amounts of GST-HIV-CA S149A (Fig 5A, compare GST-HIV CA and GST-HIV CA S149A).

To directly test the ability of MELK to phosphorylate Ser-149 in CA, we generated rabbit polyclonal antibodies that recognize only phosphorylated Ser-149 in CA (CA-S149p). Fig 5B shows that phosphorylation of virion-associated CA was undetectable with CA-S149p (top panel CA-S149p, lanes 2 and 3). We next performed an *in vitro* phosphorylation assay with envelope-stripped core prepared as in Fig 2D to determine whether MELK phosphorylates Ser-149 of CA in the multimerized viral core. Incubation of envelope-stripped core with recombinant MELK induced phosphorylation of S149 in a time-dependent manner (Fig 5B, top panel CA-S149p, lanes 3–7), which was undetectable in the presence of the immunizing peptide for CA-S149p (Fig 5B, panel Immunizing peptide [CA: 140–154 S149p]). This indicates that CA-S149p specifically detects phosphorylation of S149 in CA, which was diminished in the presence of OTSSP167 (Fig 5B, panels OTSSP167 [100 nM] and OTSSP167 [500 nM]) or calf intestinal alkaline phosphatase (Fig 5B, bottom panel CIAP, CA-S149p). To further investigate the phosphorylation of Ser-149 by MELK *in vivo*, we performed Phos-tag analysis to detect any mobility shift of phosphorylated proteins using Manganese^2+^-phos-tag SDS-PAGE [51] (Fig 5C and 5D). Migration of CA was faster from MELK-KD MT4C5 cells than from control (Non-T) cells, indicating that MELK phosphorylates CA in HIV-1-infected cells (Fig 5C, top panel CA, compare lanes 2 and 3). Treatment of cell lysate with calf intestinal alkaline phosphatase (CIAP) further down-shifted the bands (Fig 5C, top panel CA, lanes 4 and 5). In addition, essentially no difference in the mobility shift was observed with CA-S149A in Non-T and MELK-KD cells, suggesting that the Ser-149 residue is the sole phosphorylation target of MELK (Fig 5D, top panel CA, compare lanes 2 and 3). Collectively, these results clearly indicate that MELK regulates optimal capsid disassembly and efficient viral cDNA synthesis in target cells through phosphorylation of Ser-149 in CA. In fact, according to the HIV Sequence Compendium 2016 published by Los Alamos National Laboratory, the Ser-149 residue in CA is highly conserved among HIV-1 strains, suggesting its important role in HIV-1 replication (S12 Fig).

### Forced expression of MELK leads to premature HIV-1 capsid disassembly and viral cDNA synthesis in target cells

**Fig 6:**
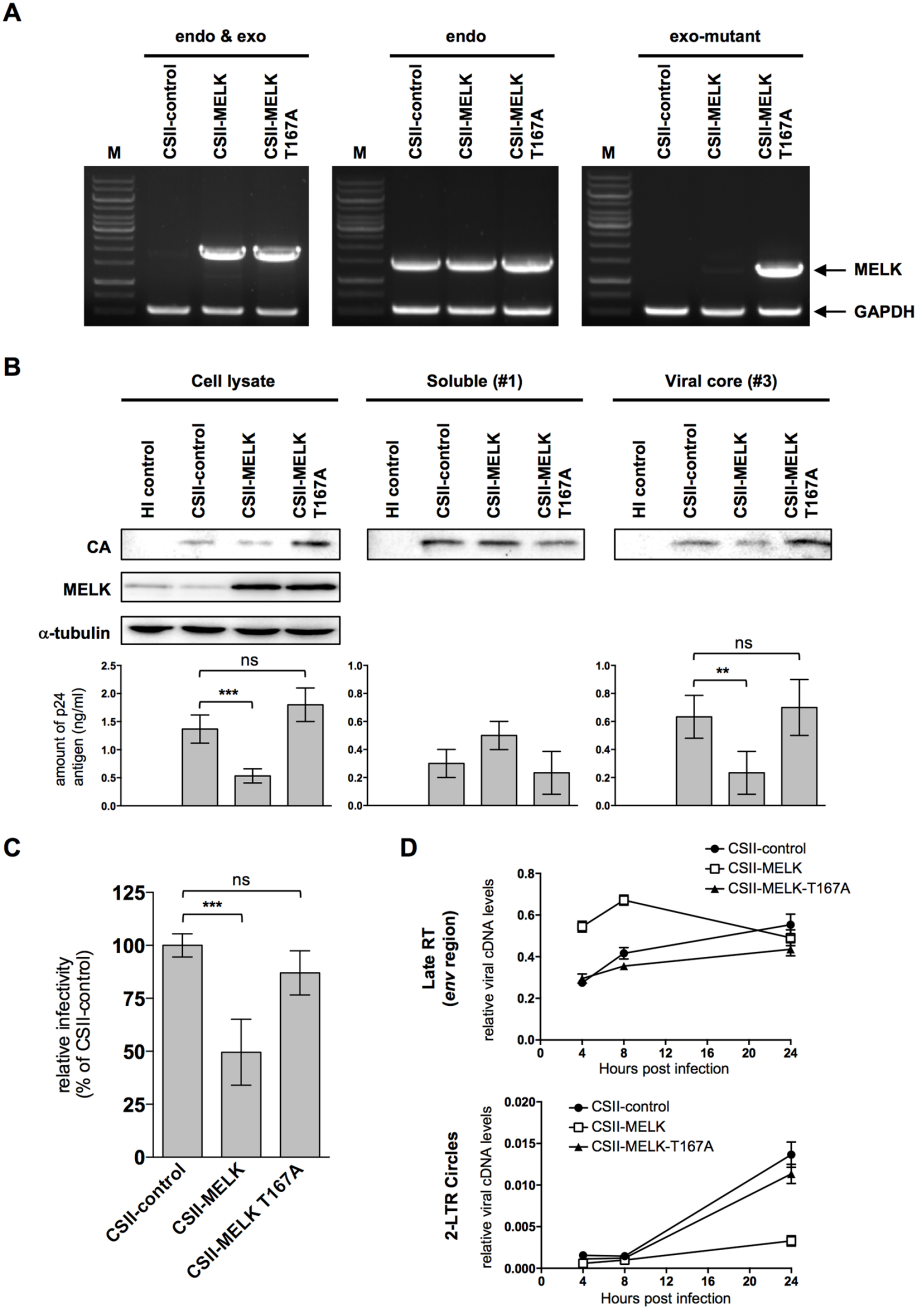
Forced expression of MELK affects the disassembly of HIV-1 core and infectivity. **(A)** Semi-quantitative RT-PCR analyses of *MELK* mRNA expression. Total RNA was prepared from Non-T MT4C5 cells transduced with empty lentivirus vector (CSII-control), MELK expression vector (CSII-MELK) or MELK mutant expression vector (CSII-MELK T167A). Total *MELK* mRNAs (left panel), endogenous *MELK* mRNA (middle panel) and exogenous mutant *MELK* mRNA (right panel) were quantitated by RT-PCR amplification with specific primer sets (MELK). A primer set for amplification of *GAPDH* mRNA was included in each reaction as an internal control (GAPDH). Experiments were performed three times and one set of representative data is shown. **(B)** Fate-of-capsid assays. Non-T MT4C5 cells transduced with empty lentivirus vector (CSII-control), MELK expression vector (CSII-MELK) or MELK mutant expression vector (CSII-MELK T167A) were infected with NL4-3 for 8 h. HIV-1 stock inactivated by incubation at 65°C for 30 min was used as a negative control (HI control). Cell lysates were prepared and analyzed as in Fig 2I. Aliquots of input, soluble fraction #1 and viral core fraction #3 were processed for immunoblotting with anti-p24 antibody (CA). Input cell lysates were also analyzed by immunoblotting with anti-MELK (MELK) and anti-alpha-tubulin antibodies (α-tubulin). Experiments were performed five times and one representative set of data is shown. The amount of CA in each fraction was quantified by HIV-1 p24 ELISA (lower panels). Error bars indicate the standard deviations calculated from five independent experiments. **(C)** Non-T MT4C5 cells transduced with empty lentivirus vector (CSII-control), MELK expression vector (CSII-MELK) or MELK mutant expression vector (CSII-MELK T167A) were infected with VSV-G-env-pseudotyped NL4-3luc. Relative luciferase activities are shown as ratios (%) of the RLU of control Non-T MT4C5 cells with standard deviations calculated from five independent experiments. **(D)** Quantitative DNA-PCR analyses of viral cDNA metabolism after HIV-1 infection of MT4C5 cells. Total DNA was extracted from the indicated cells and analyzed for the amounts of late RT product (upper panel) and 2-LTR circle form (lower panel) as in Figs 1E and 4C. Experiments were performed at least three times and error bars are standard deviations calculated from three independent experiments. The ratios of each viral cDNA level to beta-globin DNA level are given. Statistical significance was determined by one-way analysis of variance (ANOVA) with Dunnett’s multiple comparison test **(B** and **C)**. ns, not significant (*P*>0.05); *P<0.05, **P<0.01, ***P<0.001.

Our observation that constitutive phosphorylation of Ser-149 caused premature capsid disassembly and early viral cDNA synthesis but failed to support nuclear import of viral DNA suggests that a well-ordered phosphorylation of Ser-149 during CA disassembly is required for optimal uncoating, viral cDNA synthesis and nuclear import. We next examined whether forced expression of MELK in MT4C5 cells affects HIV-1 infection. Expression of exogenous and endogenous *MELK* mRNA was monitored by RT-PCR (Fig 6A). To assess how MELK overexpression influences the kinetics of capsid disassembly, we performed a fate-of-capsid assay in control (CSII-control), MELK-expressing (CSII-MELK) and catalytically inactive MELK-expressing (CSII-MELK T167A) MT4C5 cells. Overexpression of wild-type MELK resulted in an obvious decrease in pelletable CA (Fig 6B, right panels, compare CSII-control and CSII-MELK), whereas the catalytically inactive MELK rather increased them. This indicates that the catalytic activity of MELK controls CA disassembly (Fig 6B, right panels, compare CSII-control and CSII-MELK T167A). Overexpression of MELK but not the MELK mutant inhibited HIV-1 infection (Fig 6C, compare CSII-control, CSII-MELK and CSII-MELK T167A) and resulted in aberrant viral cDNA synthesis quite similar to the S149E CA mutant (Fig 6D, compare CSII-Control, CSII-MELK and CSII-MELK-T167A). These results strongly suggest that optimal phosphorylation of CA by MELK is required for efficient HIV-1 infection at the early stages.

### MELK inhibitor suppresses HIV-1 replication

**Fig 7:**
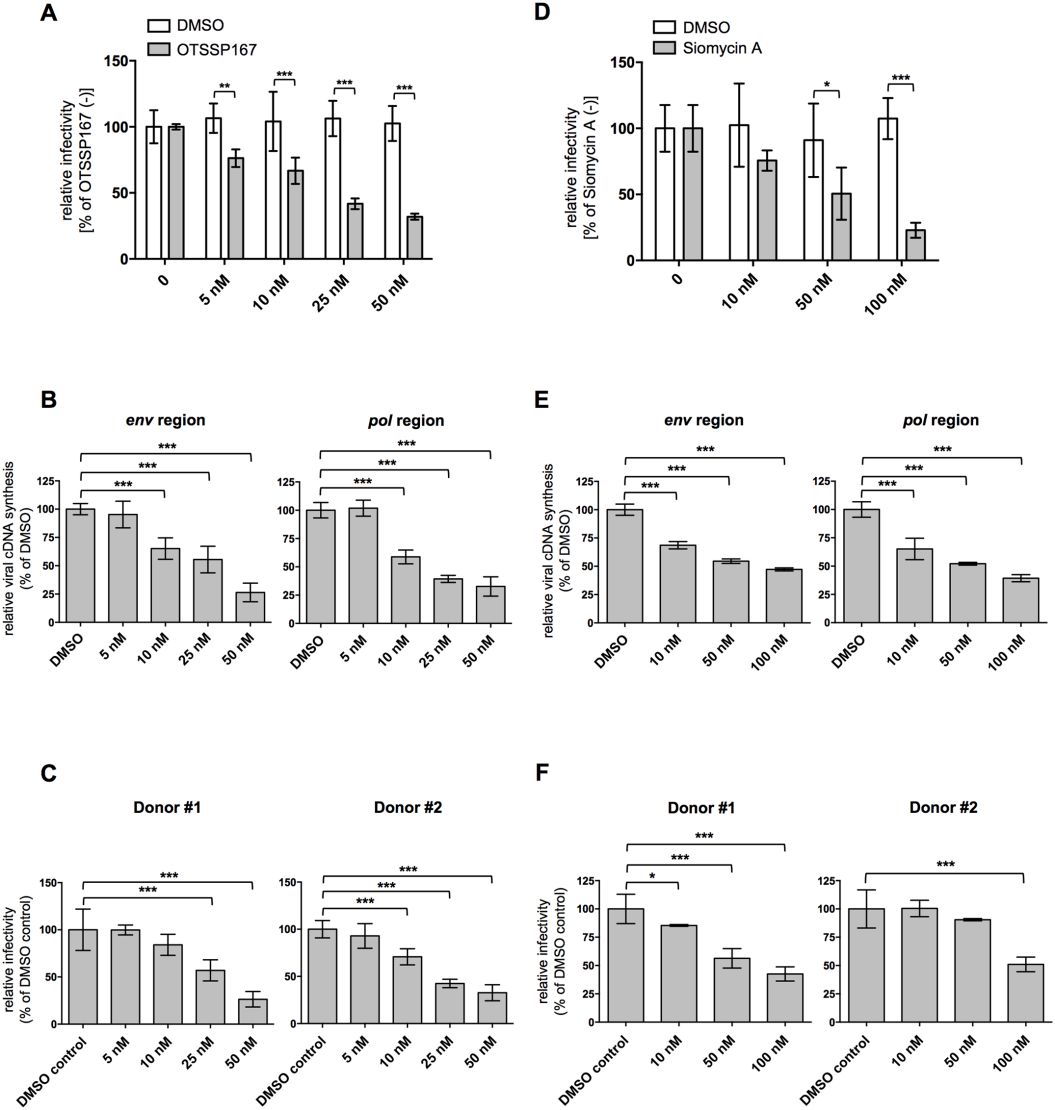
MELK inhibitor blocks HIV-1 infection in human cells. **(A)** Effect of OTSSP167 on HIV-1 single-round infection. MT4C5 cells were infected with VSV-G-pseudotyped NL4-3luc in the presence of increasing amounts of OTSSP167. **(B)** Quantitative DNA-PCR analysis of viral cDNA metabolism after HIV-1 infection in OTSSP167-treated MT4C5 cells. Total DNA from MT4C5 cells treated with OTSSP167 was extracted 8 h after HIV-1 infection and analyzed for the amount of late RT products with a primer set recognizing the *env* (left panel) or *pol* (right panel) regions. The ratios of each viral cDNA level to beta-globin DNA level are given. **(C)** Effect of OTSSP167 on single-round HIV-1 infection of PHA-stimulated PBMCs. PHA-stimulated PBMCs were infected with VSV-G-pseudotyped NL4-3luc in the presence of increasing amounts of OTSSP167. **(D)** Effect of Siomycin A on HIV-1 single-round infection. MT4C5 cells were infected with VSV-G-pseudotyped NL4-3luc in the presence of increasing amounts of Siomycin A. **(E)** Quantitative DNA-PCR analysis of viral cDNA metabolism after HIV-1 infection in Siomycin A-treated MT4C5 cells. Total DNA from MT4C5 cells treated with Siomycin A was extracted and analyzed as in **(B)**. **(F)** Effect of Siomycin A on single-round HIV-1 infection of PHA-stimulated PBMCs. PHA-stimulated PBMCs were infected with VSV-G-pseudotyped NL4-3luc in the presence of increasing amounts of Siomycin A. Error bars indicate the standard deviations calculated from five independent experiments. Statistical significance was determined by two-way analysis of variance (ANOVA) with Sidak’s multiple comparison test **(A** and **D)**, or one-way ANOVA with Dunnett’s multiple comparison test **(B**, **C**, **E** and **F)**. *P<0.05, **P<0.01, ***P<0.001.

We assessed whether OTSSP167 affects HIV-1 replication in MT4C5 cells. Single-round infection assays revealed that viral infectivity in the presence of OTSSP167 was substantially reduced in a dose-dependent manner (Fig 7A). OTSSP167 compromised viral cDNA synthesis in a dose-dependent manner (Fig 7B) and also reduced viral infectivity in PHA-stimulated peripheral blood mononuclear cells (PBMCs) derived from two healthy donors (Fig 7C).

Similar results were obtained with the macrocyclic thiazole antibiotic Siomycin A (Fig 7D–7F), initially identified as an inhibitor of the transcription factor FOXM1b and thereafter reported to reduce MELK expression in brain tumor stem-like cells *in vitro* [52–54]. MELK expression was reduced by Siomycin A in a dose-dependent manner (S13A Fig), while there was no effect on *MELK* mRNA levels in MT4C5 cells (S13B Fig). HIV-1 replication in MT4C5 cells with the replication-competent NL4-3 virus in the presence of Siomycin A was markedly inhibited in a dose-dependent manner (S13C Fig). These findings suggest that a small-molecule inhibitor of MELK has potential as an anti-HIV lead compound.

## Discussion

The main finding of the present study is that MELK regulates CA disassembly to promote viral cDNA synthesis through the phosphorylation of Ser-149 in CA during the early stages of HIV-1 infection. MELK depletion did not significantly alter the efficiency of HIV-1 entry (Fig 2A–2C), but did impair viral cDNA synthesis in association with a significant delay of CA disassembly during the early stages of HIV-1 infection (Figs 1E, 2I and 2J). Although MELK was identified based on HIV-1 infection-resistant cells during a spreading-infection, the inhibition by MELK-depletion in single-round infection assays was not complete (Fig 1C). This seems to be due, at least in part, to the difference in MOI between single-round infection assays (MOI, 1) and the initial genome-wide RNAi screen (MOI, 0.01). The different level of inhibitory effects shown in the uncoating (~2 fold) and infectivity (~10 fold) results may be explained by the widely observed finding that changes in uncoating influence reverse transcription and nuclear translocation, thereby amplifying the effect. The marked suppression of the spread of HIV-1 infection in MELK-depleted cells further suggests a role for MELK in other stages of virus replication. This is currently under investigation. The direct interaction of MELK with envelope-stripped core, but not with the monomeric form of CA, suggests an association between the host cell MELK and the incoming viral core in the cytoplasm (Fig 2D–2F). Moreover, the results in Fig 3D indicate that the env-stripped HIV-1 core is a much better substrate of MELK than GST-HIV-CA. Host cell core-binding factors such as MELK that accelerate CA dissociation from the viral core, and those that stabilize the core, such as CPSF6 [15–17], are likely required to achieve optimal stability of the viral core which is necessary for efficient viral cDNA synthesis in target cells. We further document, for the first time, phosphorylation of S149 in the multimerized viral core by MELK, and provide compelling evidence of *in vivo* phosphorylation of S149 (Fig 5B). *In vitro* phosphorylation assays have shown that the HIV-1 CA is a substrate of MELK (Fig 3C) and that Thr-119, Ser-146, Thr-148, and Ser-149 are the candidate phosphorylation targets (Fig 3F). Ser-146, Thr-148 and Ser-149 are located in the flexible linker region that may allow movement of the C-terminal domain (CTD) relative to the N-terminal domain (NTD) [55–57], whereas Thr-119 is located in the amino-terminal domain of CA. The restoration of viral cDNA synthesis in MELK-KD cells by the S149E mutation is consistent with a previous report that the flexible linker region has a critical role in optimal core stability and efficient HIV-1 replication [23]. The S149E mutation caused an earlier peak and subsequent reduction of viral cDNA synthesis in control cells as in MELK-KD cells (S9D Fig). Infection of cells overexpressing MELK with wild-type HIV-1 resulted in premature CA disassembly and aberrant viral cDNA synthesis (Fig 6B and 6D). These results collectively indicate that unusual phosphorylation of S149 in CA by MELK misguides capsid disassembly and viral cDNA synthesis (Figs 4 and 6 and S9D Fig). HIV-1 with T119E, S146E or T148E mutations yielded less late RT product in MELK-KD cells than in control Non-T cells probably because in these mutants S149 remains intact (S9A–S9C Fig). They produced late RT products in control Non-T cells much less efficiently than did wild-type so that the proportional reduction in the late RT product was not as marked as in the wild-type (S9A–S9C Fig). This reduction indicates that these mutants are sensitive to MELK depletion and that the essential target of MELK is not T119, S146 or T148. T119E and S146E mutants produced a little more late RT product than did the wild-type in MELK-KD cells (Fig 4B) and eventually failed to increase or retain this at 24 h post-infection, suggesting that these mutations promoted reverse transcription independently of MELK (Fig 4B). A previous report showed that the CA mutations E128A/R132A increased the stability of the viral core and impaired viral cDNA synthesis, while CA mutations Q63A/Q67A accelerated CA disassembly and viral cDNA synthesis, but severely impaired viral infectivity [6]. This appears consistent with our results that MELK depletion caused both the delay of CA disassembly and reduction in viral cDNA synthesis in target cells. We showed here that the S149E mutation, like Q63A/Q67A mutations, accelerated CA disassembly and viral cDNA synthesis, and severely impaired nuclear import of the viral cDNA (Fig 4). This suggests that successful viral cDNA synthesis may largely depend on the S149 phosphorylation-triggered CA disassembly and that appropriate timing and perhaps location of CA disassembly is necessary for the efficient nuclear import of viral cDNA. Indeed, previous reports showed that HIV-1 CA interacts directly with the nuclear pore complex (NPC) by binding to the cyclophilin-like domain of Nup358 [58, 59]. It is plausible that aberrant disassembly of S149E CA potentially at an inappropriate location renders recognition of CA by Nup358 difficult. The rapid decline in the amount of viral cDNA in S149E mutant-infected cells (Fig 4A–4C and S9D Fig) also suggests that premature uncoating promotes viral DNA degradation in the cytoplasm. Poor production of the S149E mutant virus (S10A and S10B Fig) may further explain why this residue has to be phosphorylated after entry. MELK-mediated phosphorylation of Ser-149 after entry may have evolved to optimize production of infectious virions and achieve an ordered CA disassembly and efficient nuclear entry. In terms of the coupled RT and capsid disassembly, it is tempting to postulate that phosphorylation of CA leads to a conformational change that unlocks the initiation of reverse transcription. This would in turn influence the activities of cytoplasmic factors involved in the regulation of capsid disassembly, such as MELK, and thereby further promote disassembly of the core.

The poor ability of the VSVG/NL4-3luc CA-S149A virus to infect control MT4C5 cells differs from a previous observation that VSVG-pseudotyped env-deleted HIV-1 CA-S149A mutants can infect LuSIV, TZM-bl and MAGIC-5B HeLa cells. This may be due in part to the different reporter systems and cell lines used. Our observation that alanine substitution of Ser-149 (S149A) ablated CA phosphorylation *in vitro* by MELK suggests that the Ser-149 residue is the sole phosphorylation target of MELK in CA (Fig 5A). The lack of difference in mobility shift of S149A CA in Non-T and MELK-KD cells shown in the Phos-tag assay further reinforces this notion (Fig 5D). A previous report showed that phosphorylation of three serine residues (Ser-109, Ser-149, and Ser-178) in CA is required for efficient reverse transcription and uncoating [19]. We therefore generated S109E and S178E mutant viruses, and found that this failed to support viral cDNA synthesis in both Non-T and MELK-KD cells, suggesting that MELK is not involved in phosphorylation of Ser-109 or Ser-178 (S9E and S9F Fig). Our Phos-tag result that treatment of lysates from HIV-1-infected MELK-KD cells with CIAP further down-shifted the CA bands suggests that CA is phosphorylated by other cellular kinases in HIV-1-infected cells (Fig 5C, top panel CA, compare lanes 3 and 5). Our results from S149E mutation experiments and overexpression of MELK regarding effects on viral DNA synthesis and nuclear import strongly suggest that phosphorylation of S149 under temporally and spatially appropriate conditions is important for enabling HIV-1 to proceed through the early stages of infection. OTSSP167 inhibits the catalytic activity of MELK while Siomycin A reduces its expression, both of which interfere with the function of MELK as a kinase and similarly reduce the infectivity of HIV-1 in PBMC (Fig 7). This decreases the likelihood of off-target effects of these drugs.

In conclusion, the essential role of MELK at an early stage of HIV-1 infection exemplifies another aspect of the functional links between viral capsid disassembly, cDNA synthesis and nuclear import. These findings contribute to our understanding of early viral life-cycle events and raise the possibility of developing a new class of anti-HIV agents targeting viral capsid disassembly.

## Materials and methods

### Cells

HEK293 (Invitrogen Corp., Carlsbad, CA), HEK293T (Invitrogen Corp., Carlsbad, CA), HeLa (ATCC) and TZM-bl [National Institutes of Health (NIH) AIDS Research and Reference Reagent Program] cells were propagated in Dulbecco’s modified Eagle medium containing 10% fetal bovine serum (FBS) and penicillin/streptomycin. MT4C5 (kindly provided by Tetsuro Matano’s lab in National Institute of Infectious Diseases, Japan) cells were maintained in complete RPMI 1640 medium supplemented with 10% FBS and penicillin/streptomycin. LuSIV cells (NIH AIDS Research and Reference Reagent Program) were cultured in RPMI 1640 supplemented with 10% FBS, penicillin/streptomycin, and 300 μg per ml hygromycin B. Phytohemaggulutinin (PHA)-activated PBMCs (PHA-PBMCs) were cultured in RPMI 1640 containing 10% FBS, penicillin/streptomycin, and 100 U IL-2 per ml. CD3/CD28-stimulated peripheral blood lymphocytes (PBL) were prepared using human T-Activator CD3/CD28 Dynabeads (Thermo Fisher Scientific, Waltham, MA) and cultured in RPMI 1640 containing 10% FBS, penicillin/streptomycin, and 100 U IL-2 per ml.

### Pharmaceuticals

Nevirapine (NVP) and Azidothymidine (AZT) were obtained from National Institutes of Health (NIH) AIDS Research and Reference Reagent Program. AMD3100 and MG132 were obtained from Sigma-Aldrich. Siomycin A was obtained from Bioaustralis. OTSSP167 was obtained from Selleck chemicals.

### Preparation of virus stocks

HEK293T cells cultured in a 10 cm dish were cotransfected with 8 μg of pNL4-3luc (*env-/nef-*) plus 2 μg of pHCMV-G (VSV-G) or pLET (HIV-1_LAI/IIIB_ envelope), using FuGENE 6 (Roche Applied Science, Mannheim Germany) as recommended by the manufacturer. Virus stocks used for analysis of viral replication were prepared by transfection of HeLa cells using LipofectAMINE LTX PLUS (Invitrogen Corp., Carlsbad, CA) with molecular clone DNAs of HIV-1_NL4-3_, pNL4-3 [60] or pNL4-3 glutamate mutants. Viruses were harvested 48 h post-transfection and filtered through a 0.45 μm syringe filter. Titers of the virus stocks were determined by their reverse transcriptase (RT) activity. The amount of CA was quantified by HIV-1 CA (p24) enzyme-linked immunosorbent assay (ZeptMetrix Corporation, Buffalo, NY). For production of lentivirus vectors, HEK293T cells were co-transfected with a lentivirus vector plasmid, HIV-1 helper virus plasmid (pCMV delta R8.2), and VSV-G protein-expression plasmid (pHCMV-G) using FuGENE6 transfection reagent. Culture supernatant of transfected HEK293T cells was collected 48 h after transfection and filtered through a 0.45 μm syringe filter. For production of retrovirus vectors, Plat-E cells were cotransfected with the retrovirus transfer plasmid pMX-luc [61] and VSV-G using FuGENE 6 transfection reagent. Culture supernatant of Plat-E cells was collected 60 h after transfection and filtered through a 0.45 μm-pore syringe filter.

### Plasmids

pNL4-3_T119E_, pNL4-3_S146E_, pNL4-3_T148E_, and pNL4-3_S149E_ were generated by site-directed mutagenesis using PrimeSTAR Mutagenesis Basal kit (TAKARA BIO Inc., Shiga, Japan) on pCR2.1 (Invitrogen Corp., Carlsbad, CA) using the NL4-3 *gag* gene (nt 689–2225) of the HIV-1_NL4-3_ genome (GenBank accession number M19921) as the template. Primers T119E-5 (5’-TGGATGGAGCATAATCCACCTATCCCA-3’) and T119E-3 (5’-ATTATGCTCCATCCATCCTATTTGTTC-3’) were used for the T119E substitution, primers S146E-5 (5’-ATGTATGAGCCTACCAGCATTCTGGAC-3’) and S146E-3 (5’-GGTAGGCTCATACATTCTTACTATTTT-3’) for the S146E substitution, primers T148E-5 (5’-AGCCCTGAGAGCATTCTGGACATAAGA-3’) and T148E-3 (5’-AATGCTCTCAGGGCTATACATTCTTAC-3’) for the T148E substitution and primers S149E-5 (5’-CCTACCGAGATTCTGGACATAAGACAA-3’) and S149E-3 (5’-CAGAATCTCGGTAGGGCTATACATTCT-3’) for the S149E substitution. These mutated DNA fragments were inserted into the *Bss*H II and *Apa* I sites of pNL4-3. The resultant plasmids are referred to as pNL4-3_T119E_, pNL4-3_S146E_, pNL4-3_T148E_, and pNL4-3_S149E_. For construction of Strep-tag II fusion-MELK, -Cyclophilin A (CypA), -rhesus monkey Trim5α (rhT5α) and Green Fluorescent Protein (GFP) expression vectors, the cDNA of human MELK was amplified by RT-PCR using MT4C5 cell-derived total RNA as the template and the cDNAs of CypA, rhT5α, and GFP were amplified by PCR using pcDNA-HA-CypA [62], pRhT5α [63], and pMax-GFP (Lonza Japan Ltd., Tokyo, Japan) as the template, respectively. Primers huMELK-5 (5’-GGGGTACCAAAGATTATGATGAACT-3’) and huMELK-3 (5’-CCGCTCGAGTTATACCTTGCAGCTAGATA-3’) were used for MELK amplification; primers pCAG-OSF-CypA-5 (5’-GGGGTACCATGGTCAACCCCACCGT-3’) and pCAG-OSF-CypA-3 (5’-CCGCTCGAGTTATTCGAGTTGTCCACAGT-3’) for CypA amplification, primers *Kpn*I-Kz-RhT5α-F (5’-GCGGTACCGCCACCATGGCTTCTGGAATCCTGCTT-3’) and *Xho*I-RhT5a-R (5’-CGCTCGAGAGAGCTTGGTGAGCACAGAG-3’) for rhT5α amplification, primers *Kpn*I-GFP-F (5’-GCGGTACCGTGAGCAAGGGCGAGGAG-3’) and *Xho*I-GFP-R (5’-CCGCTCGAGTCACTTGTACAGCTCGTCCAT-3’) for GFP amplification. The amplified cDNAs were inserted into the *Kpn* I and *Xho* I sites of pCAG-OSF or pCAG-FOS2 [64], and sequenced. The resultant plasmids are referred to as pCAG-OSF-MELK, pCAG-OSF-CypA, pCAG-FOS2-rhT5α, and pCAG-OSF-GFP. Primers huMELK-T167A-F (5’-CTACAGGCCTGCTGTGGGAGTCTGGCT-3’) and huMELK-T167A-R (5’-ACAGCAGGCCTGTAGATGGTAATCCTT-3’) were used to generate pCAG-OSF-MELK-T167A by site-directed mutagenesis. A DNA fragment encoding HIV-1 CA was amplified by PCR using pNL4-3 as the template, and were inserted into pGEX-4T-3 in frame with glutathione *S*-transferase (GST). The resulting plasmid is referred to as pGEX-HIV-CA. Primers NL43-CA-S149A-5 (5’-CCTACCGCCATTCTGGACATAAGACAA-3’) and NL43-CA-S149A-3 (5’-CAGAATGGCGGTAGGGCTATACATTCT-3’) were used to generate pGEX-HIV-CA-S149A by site-directed mutagenesis. For generation of human MELK and rhT5α lentiviral expression constructs, the cDNAs of human MELK and rhT5α were amplified by PCR using pCAG-OSF-MELK and pRhT5α as the template, respectively. Primers pENTR-MELK-5 (5’-CACCACCATGAAAGATTATGATGAACT-3’) and pENTR-MELK-3 (5’- TTATACCTTGCAGCTAGATA-3’) were used for MELK amplification. Primers RhTRIM5α-HA-F (5’-CACCACCATGGCTTCTGGAATC-3’) and RhTRIM5α-HA-R (5’-TCAAGTCTGGGACGTCGTATGGGTA-3’) were used for rhT5α amplification. Each amplified cDNA was inserted into pENTR/D-TOPO (Invitrogen Corp., Carlsbad, CA). The lentivirus vector CSII-EF-IB-RfA [65] was incubated with pENTR/D-TOPO-MELK or pENTR/D-TOPO-rhT5α-HA in the presence of Gateway LR Clonase II Enzyme Mix (Invitrogen Corp., Carlsbad, CA) according to the procedures recommended by the manufacturer. The resultant plasmids are referred to as pCSII-EF-IB-MELK or pCSII-EF-IB-rhT5α-HA. A similar approach was used to generate lentivirus vector for the T167A MELK mutant, pCSII-EF-IB-MELK-T167A.

### Establishment of an shRNA T-cell library

A puromycin-marked lentivirus vector-based shRNA library that targets over 15,000 human genes (Sigma-Aldrich, MISSION shRNA library) was used to establish shRNA-MT4C5 cell libraries. On average, there are five shRNA sequences designed for each gene target. The library was pre-divided into ten sub-pools of approximately 8,000 shRNA constructs. MT4C5 cells were transduced with the shRNA-lentivirus library and selected with puromycin (1 μg/ml) for 2 weeks.

### shRNA-based screening

Established shRNA library-expressing MT4C5 cell pools were then infected with HIV-1_NL4-3_ strain. HIV-1_NL4-3_ strain normally kills infected parental or Non-T control MT4C5 cells with a slight degree of syncytia formation, indicating effective infection-induced cell death. Two weeks after infection, cells were seeded into 96-well round-bottom cell culture plates. Several sub-pools resistant to HIV-1 infection were identified. Total cellular DNA was prepared from each sub-pool and used to detect the *pol* region of the HIV-1_NL4-3_ late reverse transcription product by quantitative PCR using TaqMan PCR (Applied Biosystems, Carlsbad, CA). Positive samples were excluded as persistently infected cells. To determine the shRNA sequences in surviving cells free from NL4-3 DNA, total cellular DNA was extracted and the DNA fragments encoding the shRNA were amplified by PCR with the primers 5’-TACAAAATACGTGACGTAGAAA-3’ and 5’-TTTGTTTTTGTAATTCTTTA-3’. The PCR products were cloned into the pCR4-TOPO vector (Invitrogen Corp., Carlsbad, CA). At least 100 PCR clones were sequenced for each surviving cell pool with the primer 5’-TTTGTTTTTGTAATTCTTTA-3’.

### Depletion of MELK in MT4C5, HEK293 and CD3/CD28-stimulated PBL

MT4C5, HEK293 and CD3/CD28-stimulated PBL were transduced with lentivirus vectors that confer puromycin resistance and express either non-targeting short hairpin RNAs (shRNA) (5’-CAACAAGATGAAGAGCACCAA-3’) (Sigma-Aldrich Co, St. Louis, MO) or those targeting human MELK (Sigma-Aldrich Co, St. Louis, MO). The shRNA targeting the 3′-UTR of MELK (5’-CTCTTAACTATGTCTCTTTGT-3’) was used to generate 293-MELK-KD-1 cells or MT4C5-MELK-KD-1 cells for reconstitution experiments; the shRNA targeting the coding sequence of MELK (5’-GCCTGAAAGAAACTCCAATTA-3’) was used to generate 293-MELK-KD-2 cells, MT4C5-MELK-KD-2 or PBL-MELK-KD-2 cells; and the shRNA targeting the coding sequence of MELK (5’-GACTAAAGCTTCACTATAATG-3’) was used to generate 293-MELK-KD-3 or PBL-MELK-KD-3 cells. Pools of cells expressing shRNA were established after selection with puromycin (2 μg/ml) for MT4C5 cells, puromycin (0.5 μg/ml) for CD3/CD28-stimulated PBL and puromycin (4 μg/ml) for HEK293 cells.

### Establishment of MT4C5 cells stably expressing MELK or a MELK mutant (T167A)

Non-T MT4C5 cells were transduced with lentivirus vectors that confer blasticidin resistance and express MELK or a MELK mutant (T167A). Transduced cell pools were established after selection with 6 μg/ml blasticidin and 2 μg/ml puromycin for 7 days.

### Reconstitution of MELK in MELK-depleted MT4C5 cells

Both Non-T MT4C5 and MELK-KD-1 MT4C5 cells established with the shRNA targeting the 3′-UTR of MELK were transduced with lentivirus vectors encoding a blasticidin resistance gene and expressing the coding region of MELK or T167A MELK mutant. Two independent pools of reconstituted cells were established for the wild-type and mutant MELK after selection with 2 μg/ml puromycin and 6 μg/ml blasticidin.

### Semi-quantitative multiplex RT-PCR

Total cellular RNA was extracted using an RNeasy Tissue Kit (QIAGEN Inc., Valencia, CA). For evaluation of *MELK* mRNA expression, semi-quantitative RT-PCR was performed with PrimeScript One-Step RT-PCR kit ver. 2.0 (TAKARA BIO Inc., Shiga, Japan). Primers 5’- ATGAAAGATTATGATGA -3’ and 5’-TTATACCTTGCAGCTAGATA-3’ were used for amplification of the entire human *MELK* coding sequence (GenBank accession number NM_014791.3). To specifically quantify endogenous human *MELK* mRNA, we used a set of primers 5’-CAAGGCAAATCATATCTTGGATCAG-3’ and 5’-GCGATCATAACAGTCTTTATGTAGG-3’ that amplify part of the coding and 3’-noncoding sequences so that exogenous *MELK* mRNA can be excluded. Primers 5’-TAACAAGGATTACCATCTACAGGCC-3’ and 5’-AATCTGACTGTGTTTGACACTTCAG-3’ were used for amplification of cDNA for the T167A MELK mutant. In each reaction tube, 1 μg of total cellular RNA and 0.4 μM of each primer were added. For standardization, glyceraldehyde-3-phosphate dehydrogenase (GAPDH)-specific primers 5’-AGGCTGGGGCTCATTTGC-3’ and 5’-GTGCTCAGTGTAGCCCAGGATC-3’ were used to quantify human *GAPDH* mRNA. PCR products were resolved on 0.8% agarose gels, visualized by ethidium bromide-staining, and the band intensity quantified by densitometric scanning.

### Measurement of viral RNA levels after viral entry

For infection, 5 × 10^5^ target MT4C5 cells were incubated for 2 h with HIV-1 stock containing 1 × 10^6^ RT counts that were pre-treated with 100 U of DNase I (Roche Applied Science, Indianapolis, IN) in the presence of 10 mM MgCl_2_ for 20 min at 37°C. DNA-free total cellular RNA was then extracted using RNeasy Mini Kits with on-column DNase digestion (QIAGEN Inc., Valencia, CA). HIV-1 stock inactivated by incubation at 65°C for 30 min was used as a negative control. Primers 5’-ATTCCTGAGTGGGAGTTTG-3’ (nt 3780–3798) and 5’-AACTTTCTATGTAGATGGGGC-3’ (nt 3863–3883) and a probe 5’- FAM-CAATACCCCTCCCTTAGTGAAGTTATGGTAC-TAMRA-3’ (nt 3800–3830) were used for amplification and detection of the *pol* region of the HIV-1_NL4-3_ virion-associated RNA by quantitative RT-PCR using TaqMan One-Step RT-PCR (Applied Biosystems, Carlsbad, CA). For standardization, a primer/probe set of the 18S ribosomal RNA was used [61]. Real-time RT-PCR was carried out in a StepOnePlus Real-Time PCR system (Applied Biosystems, Carlsbad, CA). The ratios of each viral RNA level to 18S ribosomal RNA level are given.

### Fluorescence resonance energy transfer-based HIV-1 virion fusion assay

A fusion assay was performed using HIV-1 possessing β-lactamase-Vpr chimeric proteins (BlaM-Vpr) and MT4C5-derived cells loaded with CCF2 dye, a fluorescent substrate for β-lactamase, as previously described [66]. In brief, X4 HIV-1 containing BlaM-Vpr (HIV-1_NL-E-BlaM-Vpr_) [67] was obtained by cotransfecting 293T cells with pNL-E plus pMM310 [68] encoding *Escherichia coli* β-lactamase fused to the amino terminus of Vpr [69]. MT4C5-derived cells (1×10^6^) were infected with 10 or 100 ng of HIV-1_NL-E-BlaM-Vpr_ as a measured amount of p24 by spinoculation at 1200×*g* for 2 h at 25°C as previously described [70]. Thereafter, cells were washed and incubated in RPMI containing 10% heat-inactivated fetal bovine serum for 2 h at 37°C to induce viral fusion. Cells were then washed and loaded with CCF2-AM for 1 h at RT using a GeneBLAzer *In Vivo* Detection Kit (Invitrogen Corp., Carlsbad, CA). The dye-loaded cells were incubated overnight at RT and assayed by flow cytometry. Cells permissive for HIV-1 fusion were detected by their fluorescence at 447 nm after excitation with a 405-nm violet laser in a FACSCanto II. Dead cells were stained with propidium iodide and were gated out during analysis. AMD3100 was used as a control for fusion inhibition.

### Measurement of viral cDNA levels after viral entry

For infection, 5 × 10^5^ target MT4C5 cells were incubated for 4, 8 or 24 h with HIV-1 stock containing 50 ng of p24 or 7 × 10^5^ RT counts that were pre-treated with 100 U of DNase I (Roche Applied Science, Indianapolis IN) in the presence of 10 mM MgCl_2_ for 20 min at 37°C. Total cellular DNA was then extracted using a DNeasy Blood & Tissue Kit (QIAGEN Inc., Valencia, CA). HIV-1 stock inactivated by incubation at 65°C for 30 min was used as a negative control. Primers 5’-GGCTAACTAGGGAACCCACTGC-3’ (nt 496–517) and 5’-CTGCTAGAGA TTTTCCACACTGAC-3’ (nt 612–635) and a probe 5’- TAGTGTGTGCCCGTCTGTTG TGTGAC-3’ (nt 554–579) were used in real-time PCR for amplification and detection of the *R/U5* region of the HIV-1_NL4-3_ early reverse transcription product [71]; and primers 5’-ATTCCTGAGTGGGAGTTTG-3’ (nt 3780–3798) and 5’-AACTTTCTATGTAGATGGGGC-3’ (nt 3863–3883) and a probe 5’- FAM-CAATACCCCTCCCTTAGTGAAGTTATGGTAC-TAMRA-3’ (nt 3800–3830) were used for amplification and detection of the *pol* region of the HIV-1_NL4-3_ late reverse transcription product; primers 5’-CAGGAAGTAGGAAAAGCAATGT-3’ (nt 7496–7517) and 5’-CGAGATCTTCAGACCTGGA-3’ (nt 7609–7627) and a probe 5’-FAM-CCTCCCATCA GTGGACAAATTAGATGTTC-TAMRA-3’ (nt 7523–7551) were used for amplification and detection of the *env* region of the HIV-1_NL4-3_ late reverse transcription product; and primers 5’-CCCTCAGACC CTTTTAGTCAGTG-3’ (nt 9668–9690) and 5’-TGGTGTGTAGTTCTGCCAATCA-3’ (nt 77–98) and a probe 5’-FAM-TGTGGATCTACCACACACAAGGCTACTTCC-TAMRA-3’ (nt 46–75) were used for amplification of the 2-LTR circle from the HIV-1_NL4-3_ cDNA. Real-time PCR was carried out in a StepOnePlus Real-Time PCR system (Applied Biosystems, Carlsbad, CA). To determine the absolute copy numbers of viral DNA or 2-LTR circles in HIV-1 infected cells, we employed a calibration curve using the pNL4-3 or pGEM/NL-2LTR [71] serially diluted with a constant amount of whole cell DNA from uninfected cells. The absolute amount of beta-globin DNA determined in the same way was used to normalize the results, as described previously [39]. The ratios of each viral cDNA level to beta-globin DNA level are given. In the case of cells transduced with a lentivirus vector containing the *R/U5* region, amplified viral cDNA level in HIV-1-infected cells was determined after subtraction of the level in uninfected cells.

### Immunoblotting

Whole cell lysates were prepared as follows: Cells were washed once with PBS, suspended in PBS (500 μl per 1 × 10^7^ cells) and mixed with an equal volume of 2 × sample buffer (4% sodium dodecyl sulfate, 125 mM Tris-HCl, pH 6.8, 10% 2-mercaptoethanol, 10% glycerol, and 0.002% bromphenol blue). Proteins were solubilized by heating for 5 min at 95°C. Cell lysates were subjected to SDS-PAGE; proteins were transferred to PVDF membranes and reacted with a rabbit monoclonal antibody to MELK (Abcam Inc, Cambridge, MA), mouse monoclonal antibody to HIV-1 p24 (Abcam Inc, Cambridge, MA), goat polyclonal antibody to gp120 (Abcam Inc, Cambridge, MA), HIV-1-positive pooled serum from infected individuals (subtype B), mouse monoclonal antibody to FLAG (Wako Pure Chemical Industries, Ltd., Osaka, Japan), mouse monoclonal antibody to alpha-tubulin (Sigma-Aldrich Co, St. Louis, MO) or phospho-specific antibodies that recognize only phosphorylated Ser-149 in CA (CA-149p) produced by Sigma-Aldrich’s Phosphorylation-Specific Antibody Production Services (Sigma-Aldrich Co, St. Louis, MO) (see Supporting information for details).

### Analysis of HIV-1 replication in human T cells

MT4C5 cells (1 × 10^5^) were exposed to HIV-1 stock containing 10 pg of p24. Virus production was monitored for 14 days post-infection by measuring RT activity in the culture supernatants. Mean values from three independent experiments are shown.

### Single-round infection assay

Parental and shRNA-expressing MT4C5 cells (5 × 10^5^) were infected for 24 h with 10 ng (p24) of VSV-G/NL4-3luc, HIV-1env/NL4-3luc, or VSV-G/MLV-luc normalized by reverse transcriptase (RT) counts corresponding to 10 ng (p24) of VSV-G/NL4-3luc in 24-well plates. Cells were then harvested and lysed 24 h post-infection. LuSIV cells [72] (5 × 10^5^) were infected for 24 h in 24-well plates with HIV-1 CA mutants normalized by RT counts corresponding to 50 ng (p24) of HIV-1wt. Cells were then harvested and lysed 24 h post-infection. TZM-bl cells [73] (5 × 10^4^) were infected for 48 h in 48-well plates with HIV-1 CA mutants normalized by RT counts corresponding to 50 ng (p24) of HIV-1wt. Cells were then harvested and lysed 48 h post-infection. The luciferase activity was measured using the GloMAX multidetection system (Promega Corp, Madison, WI).

### Fate-of-capsid assay

The fate-of-capsid assay was performed with minor modifications [39] as previously described. Briefly, 5 × 10^6^ of 293, 293-non-target shRNA or 293-MELK-KD-3 cells were replated in a 10 cm plastic dish one day before assay. Cells were inoculated with 5 × 10^6^ RT counts of VSV-G/NL4-3luc virus. After incubation at 4°C for 30 min, cells were incubated at 37°C for 4 or 8 h. MT4C5-derived cells (5 × 10^6^) were inoculated with 2 × 10^7^ RT counts of wild-type HIV-1 (HIV-1_NL4-3_ strain) prepared in HeLa cells. After incubation at 4°C for 30 min, cells were incubated at 37°C for 2, 8 or 24 h. Cells were then washed twice with ice-cold PBS(-) containing 0.005% Trypsin/EDTA to detach virions from the cellular surface and once with ice-cold PBS(-) to remove Trypsin/EDTA. Washed cells were resuspended in 1 ml of hypotonic lysis buffer [10 mM Tris-HCl (pH 8.0), 10 mM KCl, 1 mM EDTA and protease inhibitor cocktail (NACALAI TESQUE, INC, Kyoto, Japan)] and incubated on ice for 15 min. Swollen cells were lysed in a 7 ml-Dounce homogenizer with a ‘tight’ pestle (15 gentle strokes making a half-turn of the pestle per each stroke) and cell lysates cleared by centrifugation at 2,000 × g for 3 min at 4°C. Cleared cell extracts (0.8 ml) were layered over 20%–60% sucrose cushions prepared in PBS and centrifuged at 4°C and 35,000 rpm for 70 min in a Beckman SW50.1 rotor; 50 μl of the cell extract was reserved as a ‘cell lysate’ fraction. After centrifugation, three fractions of 1.1 ml each were collected from the top of the gradient. Aliquots of each fraction of the step gradients were subsequently processed for immunoblotting. The amount of CA protein in each fraction was quantified using HIV-1 CA (p24) enzyme-linked immunosorbent assay kits (ZeptMetrix Corporation, Buffalo, NY).

### Isolation of envelope-stripped cores

Envelope-stripped HIV-1 cores were prepared as described previously [20]. Briefly, HIV-1-containing culture supernatants were prepared by transiently transfecting HeLa cells with pNL4-3 using LipofectAMINE LTX PLUS (Invitrogen Corp., Carlsbad, CA). Two ml of 20% sucrose solution was placed at the bottom of model SW55 centrifuge tubes and overlaid with 3 ml of HIV-1-containing culture supernatant described above. Samples were then centrifuged for 60 min at 35,000 rpm at 4°C. Particulate HIV-1 were resuspended in PBS(-) containing a protease inhibitor cocktail (NACALAI TESQUE, INC, Kyoto, Japan). This suspension was loaded onto the top of a discontinuous sucrose density gradient composed of 1.0 ml 30% sucrose solution at the bottom of model SW55 centrifuge tubes covered by 1.0 ml 0.1% Triton X-100 in 10% sucrose solution and then centrifuged in a model SW55Ti rotor for 120 min at 35,000 rpm at 4°C. Particulate CA protein was used for pull-down assays with Strep-tagged MELK or processed for immunoblotting using anti-p24 antibody (CA). The amount of particulate CA protein was quantified using HIV-1 CA (p24) enzyme-linked immunosorbent assay kits (ZeptMetrix Corporation, Buffalo, NY).

### Negative staining electron microscopy

Envelope-stripped HIV-1 cores isolated by ultracentrifugation were absorbed onto Formvar-coated copper grids, and stained with 2% phosphotungstic acid solution. The images were recorded with a Tecnai F20 transmission electron microscope (FEI Company, Hillsboro, OR) at 200kV.

### Affinity precipitation of HIV-1 cores with Strep-tag II fusion protein

HeLa cells were transfected with pCAG-OSF-MELK, pCAG-OSF-GFP, pCAG-OSF-CypA, or pCAG-FOS2-rhT5α, harvested 48 h post-transfection and lysed in a 7 ml-Dounce homogenizer. Cell extracts were incubated with Strep-Tactin Sepharose for 2 h at 4°C. Purified Strep-tagged protein complexes were incubated with envelope-stripped HIV-1 cores (1,000 ng p24) for 2 h at 4°C. After extensive washing, Strep-tagged protein complexes were released by boiling in SDS-PAGE loading buffer and the proteins were analyzed by 12% SDS-PAGE and Western blotting using mouse anti-FLAG antibody (FLAG) and mouse anti-p24 antibody (CA).

### Preparation of recombinant proteins and synthetic peptides

*E*.*coli* BL21 CodonPlus-RIL cells (Agilent Inc. Santa Clara, CA) transformed with pGEX-4T-3 or pGEX-HIV-CA were used for purification of GST proteins using standard methods. Fifteen independent synthetic peptides covering HIV-1 CA were designed and provided by Sigma-Aldrich (Sigma-Aldrich Co, St. Louis, MO). The amount of CA protein was quantified using HIV-1 CA (p24) enzyme-linked immunosorbent assay kits (ZeptMetrix Corporation, Buffalo, NY).

### In vitro phosphorylation assay

In vitro phosphorylation assays were performed with the ADP-Glo MELK kinase assay kit, following the manufacturer’s instructions (Promega Corp, Madison, WI). Briefly, 100 ng of recombinant activated MELK was incubated with either GST, GST-HIV-CA or Env-stripped HIV-1 core proteins ranging from 100 to 2,000 ng or synthetic peptides, ZIPtide, ranging from 500 to 2,000 ng for 60 min at 30°C in the presence of ultrapure ATP. Light emission was measured using the GloMAX multidetection system (Promega Corp, Madison, WI). ZIPtide was used as a phosphorylation standard for MELK. For the detection of phosphorylated CA protein in the multimerized viral core, 200 ng of recombinant activated MELK was incubated with envelope-stripped HIV-1 core containing 100 ng of p24 at 30°C in the presence of ultrapure ATP. The phosphorylation reaction was terminated by the addition of 2 × sample buffer. The proteins were subsequently processed for immunoblotting using rabbit polyclonal antibodies to phospho-S149-CA, mouse monoclonal antibody to HIV-1 p24 (Abcam Inc, Cambridge, MA), mouse monoclonal antibody to HIV-1 p17 (Abcam Inc, Cambridge, MA), goat polyclonal antibody to gp120 (Abcam Inc, Cambridge, MA), or rabbit monoclonal antibody to MELK (Abcam Inc, Cambridge, MA).

### Phos-tag assay

Briefly, 5 × 10^6^ Non-T or MELK-KD MT4C5 cells were inoculated together with 2 × 10^7^ RT counts of wild-type HIV-1 (HIV-1_NL4-3_ strain) or 5 × 10^6^ RT counts of VSV-G/NL4-3luc or VSV-G/NL4-3 CA-S149A viruses. After incubation at 4°C for 30 min, cells were further incubated at 37°C for 8 h. Cells were then washed twice with ice-cold PBS(-) containing 0.005% Trypsin/EDTA to remove virions from the cell surface and once with ice-cold PBS(-) to remove Trypsin/EDTA. Washed cells were resuspended in 1 ml of hypotonic lysis buffer [10 mM Tris-HCl (pH 8.0), 10 mM KCl, 1 mM EDTA, protease inhibitor cocktail (NACALAI TESQUE, INC, Kyoto, Japan) and phosphatase inhibitor cocktail (Roche Diagnostics GmbH, Mannheim, Germany)] and incubated on ice for 15 min. Swollen cells were lysed in a 7 ml-Dounce homogenizer with a ‘tight’ pestle (15 gentle strokes making a half-turn of the pestle at each stroke) and cell lysates cleared by centrifugation at 2,000 × g for 3 min at 4°C. Proteins were separated in 10% precast SDS-polyacrylamide gels prepared with 50 μM acrylamide-pendant Phos-tag ligand (Wako Pure Chemical Industries, Ltd., Osaka, Japan) and were analyzed by immunoblotting with mouse anti-p24 antibody.

### Statistical analysis

All data are obtained from at least three independent experiments. The average values are presented with error bars indicating the standard deviation (SD) and the statistical significance was analyzed using one-way analysis of variance (ANOVA) with Dunnett’s or Tukey’s multiple comparison tests, two-way analysis of variance (ANOVA) with Tukey’s or Sidak’s multiple comparison test, or Student’s *t*-test. All the statistical analyses were performed using Prism 6 software (GraphPad Software, Inc). *P* values below 0.05 (P<0.05, *; P<0.01, **; P<0.001, ***) were considered significant. Unpaired two-tailed Student’s *t*-test was used for the data shown in Fig 2B, 2C, 2H and 2J to test whether the means of the two groups were significantly different (five biological replicates). One-way analysis of variance (ANOVA) with Dunnett’s multiple comparison test was used for data in Figs 1C, 2I, 3A, 6B, 6C, 7B, 7C, 7E and 7F to determine whether the means of multiple groups were significantly different from a single group (five biological replicates). One-way ANOVA with Tukey’s multiple comparison test was used in Fig 4D to determine whether the means of four groups were significantly different from each other (five biological replicates). Two-way ANOVA with Tukey’s multiple comparison test was used in Figs 3C, 3D and 5A to determine significant difference by comparing of the means specified by two factors (five biological replicates). Two-way ANOVA with Sidak’s multiple comparison test was used in Fig 7A and 7D to determine significant difference by pairwise comparison of the means specified by two factors (five biological replicates).

## Supporting information

S1 Methods Supporting methods including additional methods. (DOCX)

S1 Table Host factors detected by Genome-wide RNAi screen in this study. Explanation of column headings in the table: *No*: Number of identified host factor in this screen; *Symbol;* gene symbol; *21 mer target sequence*: shRNA sequences identified in infection-resistant cells free from HIV-1 DNA; *GPP Clone ID*: Identification data of shRNA provided by Genetic Perturbation Platform (GPP); *GO-Biological Process*: Identified biological process based upon gene ontology; *GO-Cellular component*: Identified cellular component based upon gene ontology. Information on gene ontology shown in this table was obtained from the Gene Ontology Annotation Database (http://www.ebi.ac.uk/GOA).
(XLSX)

S1 Fig Cell surface expression of CD4 and CXCR4 molecules in parental, non-target shRNA and MELK-KD MT4C5 cells. Cells were stained with anti-CD4 (left panels) or anti-CXCR4 mAb (right panels). As controls, MT4C5 cells were stained with isotype control mAbs (top panels).
(TIFF)

S2 Fig Effect of MELK depletion on cell-cycle progression. Effects of MELK depletion on cell cycle progression were determined using propidium iodide (PI) staining and FACS analysis. Non-T and MELK-KD-2 MT4C5 cells were treated with [top and middle panels Demecolcine (+), Non-T and MELK-KD-2] or without [top and middle panels Demecolcine (-), Non-T and MELK-KD-2] 0.05 μg/ml Demecolcine (Wako Pure Chemical Industries, Ltd., Osaka, Japan) for 16 h to synchronize them in M phase. Demecolcine was then removed and the cells were cultured for 24 h with fresh growth medium, stained with PI, and analyzed by FACS [top and middle panels 1 day after washout (wo), Non-T and MELK-KD-2]. Graphs show the distribution of cells in distinct cell cycle phases from five independent experiments (bottom panels). The average percentages of cells in each cell cycle phase (SubG_0_/G_1_, G_0_/G_1_, S and G_2_/M) are shown [bottom left panel: Demecolcine (-), bottom middle panel: Demecolcine (+), bottom right panel: 1 day after wo]. Error bars are standard deviations calculated from five independent experiments. Statistical significance was determined by unpaired two-tailed Student’s *t* test. ns, not significant (*P*>0.05).
(TIFF)

S3 Fig The inhibition of HIV-1 infection by MELK depletion depends on its expression level. **(A)** Lysates of HEK293 cells stably expressing non-target shRNA or MELK-specific shRNA (MELK-KD-1, 2 and 3) were immunoblotted with anti-MELK or anti-alpha-tubulin antibodies. (**B)** Total RNA from the cells in **(A)** was extracted and examined for *MELK* mRNA expression by multiplex RT-PCR amplification (MELK). The primer set for amplification of *GAPDH* mRNA was included in each reaction as an internal control (GAPDH). **(C)** Effect of MELK depletion on single-round HIV-1 infection in HEK293 cells. HEK293 cells described in **(A)** and **(B)** were infected with VSV-G-pseudotyped NL4-3luc. The mean luciferase value from non-target shRNA HEK293 cells was arbitrarily set as 100%. Error bars reflect the standard deviations calculated from five independent experiments. **(D)** Lysates of CD3/CD28-stimulated PBLs stably expressing non-target shRNA or MELK-specific shRNA (PBL-MELK-KD-2 and 3) were immunoblotted with anti-MELK or anti-alpha-tubulin antibodies. **(E)** Total RNA was extracted and *MELK* mRNA expression determined by multiplex RT-PCR amplification (MELK). A primer set for amplification of *GAPDH* mRNA was included in each reaction as an internal control (GAPDH). **(F)** Effect of MELK depletion on a single-round of HIV-1 infection in CD3/CD28-stimulated PBL. PBL, Non-T, PBL-MELK-KD-2 and PBL-MELK-KD-3 cells described in **(D)** and **(E)** were infected with VSV-G-pseudotyped NL4-3luc. The mean luciferase value from non-target shRNA CD3/CD28-stimulated PBL was arbitrarily set as 100%. Error bars are standard deviations calculated from five independent experiments. Statistical significance was determined by one-way analysis of variance (ANOVA) with Dunnett’s multiple comparison test **(C** and **F)**. ns, not significant (*P*>0.05); *P<0.05, **P<0.01, ***P<0.001.
(TIFF)

S4 Fig Immunoblot analyses showing MELK bound to envelope-stripped cores of HIV-1. **(A)** HeLa cells were transfected with pCAG-OSF or pCAG-OSF-MELK. Transfected HeLa cell extracts were incubated with Strep-Tactin Sepharose and OSF-tagged proteins were purified. OSF-tagged control (lanes 1 and 3) and MELK (lanes 2 and 4) proteins were then incubated with purified HIV-1 virions (lanes 1 and 2) or envelope-stripped cores (lanes 3 and 4), and complex formation was assessed by immunoblotting (IB) using rabbit anti-MELK antibody (MELK) and mouse anti-p24 antibody (CA). **(B)** Purified OSF-tagged control (lane 1) and MELK (lane 2) proteins were incubated with soluble CA (input). Complex formation was assessed by immunoblotting (IB) using rabbit anti-MELK antibody (MELK) and mouse anti-p24 antibody (CA).
(TIFF)

S5 Fig Fate-of-capsid assay with replication-competent HIV-1 at 4 and 8 h post-infection. Effect of MELK depletion on the fate of the HIV-1 CA in MT4C5 cells at 4 and 8 h post-infection analyzed as in Fig 2I.
(TIFF)

S6 Fig MELK depletion causes a delay of CA disassembly in HEK293 cells. **(A)** Virion-associated viral RNA was quantified by quantitative RT-PCR 2 h after infection of Non-T or 293-MELK-KD-3 cells with wild-type HIV-1. **(B)** Effect of MELK depletion on the fate of the HIV-1 CA in HEK293 cells analyzed as in Fig 2I. **(C)** Percentage of the pelletable CA within total CA as quantified by p24 ELISA shown in **S6B Fig**. **(D)** Non-T or MELK-KD-3 HEK293 cells were infected with VSV-G-pseudotyped HIV-1 for 2, 4 or 8 h in the presence or absence of the proteasome inhibitor MG132 (0, 5, or 20 μM). Whole cell lysates were immunoblotted with anti-p24 (CA) or anti-alpha-tubulin (α-tubulin) antibodies (left panels). Experiments were performed five times and one representative set of data is shown. The amounts of CA in the whole cell lysates were quantified by HIV-1 p24 ELISA (right panels). Error bars indicate the standard deviations calculated from five independent experiments. **(E)** Results of p24 ELISA showing the steady-state levels of CA in fraction #3 in Fig 2I **and S6B Fig** at the indicated time points (MT4C5, left panel; HEK293, right panel). Statistical significance was determined by two-way analysis of variance (ANOVA) with Sidak’s multiple comparison test **(D)**, or unpaired two-tailed Student’s *t* test **(A**, **B**, and **C)**. ns, not significant (*P*>0.05); *P<0.05, **P<0.01, ***P<0.001.
(TIFF)

S7 Fig Verification of MELK expression by immunoblot or semi-quantitative RT-PCR analyses. **Panel protein:** Parental MT4C5 (lane 1), Non-T (lane 2) or MT4C5-MELK-KD-1 cells (lanes 3) transduced with control vector (lane 4) or vector for wild-type MELK (lanes 5 and 6, two independent cell pools) or catalytically inactive T167A MELK mutant (lanes 7 and 8, two independent cell pools) were used. Cell lysates were immunoblotted with anti-MELK or anti-α-tubulin antibodies. **Panel mRNA:** Total RNA from cells listed above was extracted. Total *MELK* mRNAs (upper panel), endogenous *MELK* mRNA (middle panel) and exogenous mutant *MELK* mRNA (bottom panel) were quantified by RT-PCR amplification with specific primer sets (MELK). The primer set for amplification of *GAPDH* mRNA was included in each reaction as an internal control (GAPDH). Experiments were performed three times and one set of representative data is shown.
(TIF)

S8 Fig *In vitro* luminescent kinase assay with recombinant active MELK and increasing amounts of recombinant CA protein. Phosphorylation of recombinant CA by MELK was monitored as in Fig 3C. Error bars reflect the standard deviations calculated from three independent experiments.
(TIFF)

S9 Fig Quantitative DNA-PCR analyses of viral cDNA metabolism after HIV-1 infection of MT4C5 cells. **(A-F)** Total DNA was extracted from non-target shRNA (Non-T) or MELK-depleted (MELK-KD-2) MT4C5 cells at the indicated time points (4, 8 and 24 h) after wild-type or indicated mutants of HIV-1 infection and analyzed for the amounts of late RT product containing the *env* region. Experiments were performed at least three times and error bars are standard deviations calculated from three independent experiments. The ratios of each viral cDNA level to beta-globin DNA level are given. **(G)** Quantitative RT-PCR analyses of virion-associated viral RNA at 2 h after infection of Non-T or MELK-KD-2 MT4C5 cells with wild-type HIV-1 or CA S149E HIV-1 mutant. Error bars indicate the standard deviations calculated from five independent experiments. Statistical significance was determined by unpaired two-tailed Student’s *t* test **(G)**. ns, not significant (*P*>0.05); *P<0.05, **P<0.01, ***P<0.001.
(TIFF)

S10 Fig Phenotypic characterization of Ser/Glu or Thr/Glu HIV-1 CA mutants. **(A, B)** Virus production was monitored by assessing RT activity **(A)** or p24 antigen **(B)** in culture supernatants of HeLa cells. **(C)** Viral infectivity was evaluated by infection of TZM-bl (upper panel) and LuSIV (lower panel) indicator cell lines with culture supernatants shown in **(A)** normalized by RT activity. Relative luciferase activities are shown as percentages (%) of that of NL4-3wt with standard deviations calculated from five independent experiments.
(TIFF)

S11 Fig Phenotypic characterization of Ser/Ala HIV-1 CA (S149A) mutants. **(A)** MT4C5, Non-T and MELK-KD-2 cells were infected with VSV-G-env-pseudotyped NL4-3luc CA-wt or CA-S149A normalized by reverse transcriptase (RT) counts corresponding to 10 ng (p24) of VSV-G/NL4-3luc CA-wt. Relative luciferase activities are shown with standard deviations calculated from five independent experiments. **(B)** The CA-S149A results in each cell pool were compared on the same Y axis setting. Statistical significance was determined by unpaired two-tailed Student’s *t* test **(A)**, or one-way analysis of variance (ANOVA) with Dunnett’s multiple comparison test **(B)**. ns, not significant (*P*>0.05); *P<0.05, **P<0.01, ***P<0.001.
(TIFF)

S12 Fig Alignment of the amino acid sequences of the inner region of HIV-1 capsid (from the 120th to 152nd amino acid position) of the known HIV-1 subtypes. The sequences are aligned with the HIV.HXB2 sequence. The arrow indicates the 149th amino acid from the N-terminus of HIV-1 capsid. Dashes indicate amino acid sequence identity.
(TIFF)

S13 Fig Effects of Siomycin A on HIV-1 replication in human T cells. **(A)** Immunoblot analyses with anti-MELK (upper panel) or α-tubulin (lower panel) monitoring endogenous MELK expression in MT4C5 cells treated with increasing amounts of Siomycin A. **(B)** Semi-quantitative RT-PCR analysis of *MELK* and *GAPDH* mRNA expression in MT4C5 cells described in **(A)**. **(C)** Effect of Siomycin A on HIV-1 replication in MT4C5 cells. The virion-associated RT activity was monitored at the indicated time points in culture supernatants of MT4C5 cells treated with Siomycin A (10 nM: open circles, 50 nM: closed triangles, 100 nM: open diamonds) and those of MELK-KD-2 (closed diamonds). Error bars reflect the standard deviations calculated from three independent experiments.
(TIFF)
