# Supplementary material for: Phosphorylation of the HIV-1 capsid by MELK triggers uncoating to promote viral cDNA synthesis
Source: PLoS Pathog. 2017 Jul 6;13(7):e1006441. doi: 10.1371/journal.ppat.1006441 (PMC5500366; doi:10.1371/journal.ppat.1006441)
Supplement: S1 Methods — (DOCX) [file ppat.1006441.s001.docx]

**S1 Methods. Supporting methods including additional methods**

**Generation of phospho-specific antibodies that recognize only phosphorylated Ser-149 in CA (CA-S149p)**

CA-S149p antibodies were prepared by Sigma-Aldrich’s Phosphorylation-Specific Antibody Production Services (Sigma-Aldrich Co, St. Louis, MO). Briefly, a synthetic phosphopeptide corresponding to the phosphorylated Ser-149 residue covering 140-154 aa in the HIV-1_NL4-3_ CA protein (HIV-1 CA 140-154 149pS) was designed, provided by Sigma-Aldrich (Sigma-Aldrich Co, St. Louis, MO) and used to immunize rabbits. Rabbit serum was obtained 11-weeks after immunization with keyhole limpet hemocyanin (KLH)-conjugated phosphopeptide. To purify the resulting antisera, a nonphosphorylated peptide (HIV-1 CA 140-154) linked to Sepharose was used to remove antibodies that bound the nonphosphorylated HIV-1 CA protein. This was followed by affinity enrichment of antibodies binding the phosphopeptide (HIV-1 CA 140-154 149Sp) linked to Sepharose, which were then shown to bind the phosphorylated Ser-149 in HIV-1 CA protein. The antibody response to the phosphopeptide was assayed and verified via ELISA with the phosphopeptide or nonphosphopeptide immobilized on a substrate plate.

**Flow Cytometric Analysis for detection of CD4 and CXCR4 expression on the cell surface of parental, Non-T and MELK-KD MT4C5 cells**

Cells were stained with FITC-conjugated anti-human CD4 (BioLegend, San Diego, CA) or PerCP/Cy5.5-conjugated anti-human CD184 (CXCR4) (BioLegend, San Diego, CA) and analyzed by BD FACS Calibur (BD Biosciences, San Jose, CA). As isotype controls, FITC-conjugated anti-mouse IgG2b (BioLegend, San Diego, CA) and PerCP/Cy5.5 anti-mouse IgG2a (BioLegend, San Diego, CA) were used.

**Preparation of the GST-free recombinant HIV-1 CA protein**

GST-tagged HIV-1 CA protein prepared with pGEX-HIV-CA was incubated with Thrombin-agarose (Sigma-Aldrich Co, St. Louis, MO) to cleave the protein to GST and HIV-1 CA. The mixture was then centrifuged for 5 min at 500 × *g* at 4℃ to obtain the supernatant containing the cleaved fusion protein. The supernatant was then incubated with Sepharose GL4B beads (GE Healthcare Bio-Sciences, Pittsburgh, PA). The cleared supernatant containing GST-free HIV-1 CA protein was collected by centrifugation at 500 × *g* for 5 min at 4℃.
