# Supplementary figures and images for: Phosphorylation of the HIV-1 capsid by MELK triggers uncoating to promote viral cDNA synthesis
Source: PLoS Pathog. 2017 Jul 6;13(7):e1006441. doi: 10.1371/journal.ppat.1006441 (PMC5500366; doi:10.1371/journal.ppat.1006441)

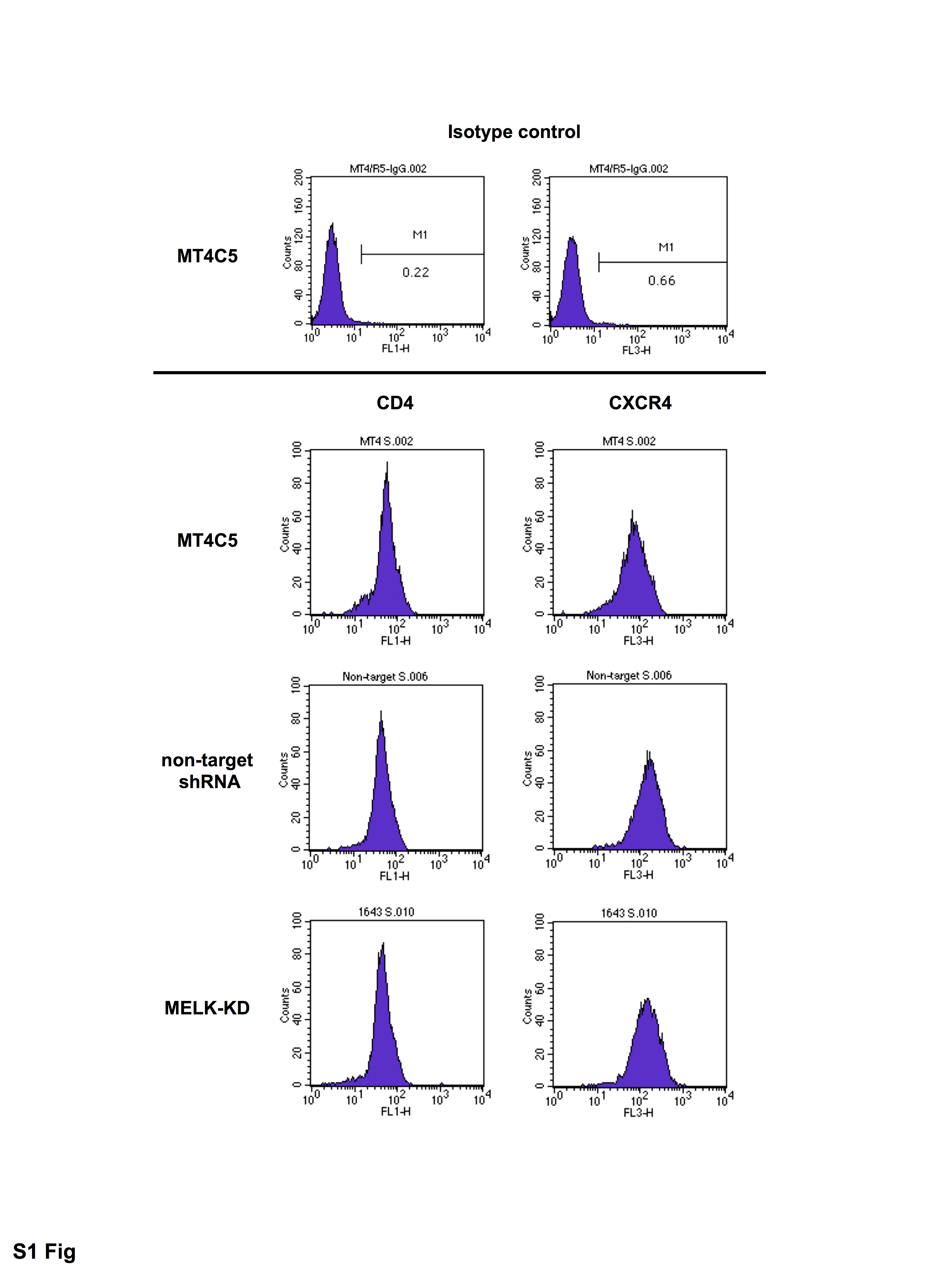

Supplement: S1 Fig — Cells were stained with anti-CD4 (left panels) or anti-CXCR4 mAb (right panels). As controls, MT4C5 cells were stained with isotype control mAbs (top panels). (TIFF) [file ppat.1006441.s003.tiff]

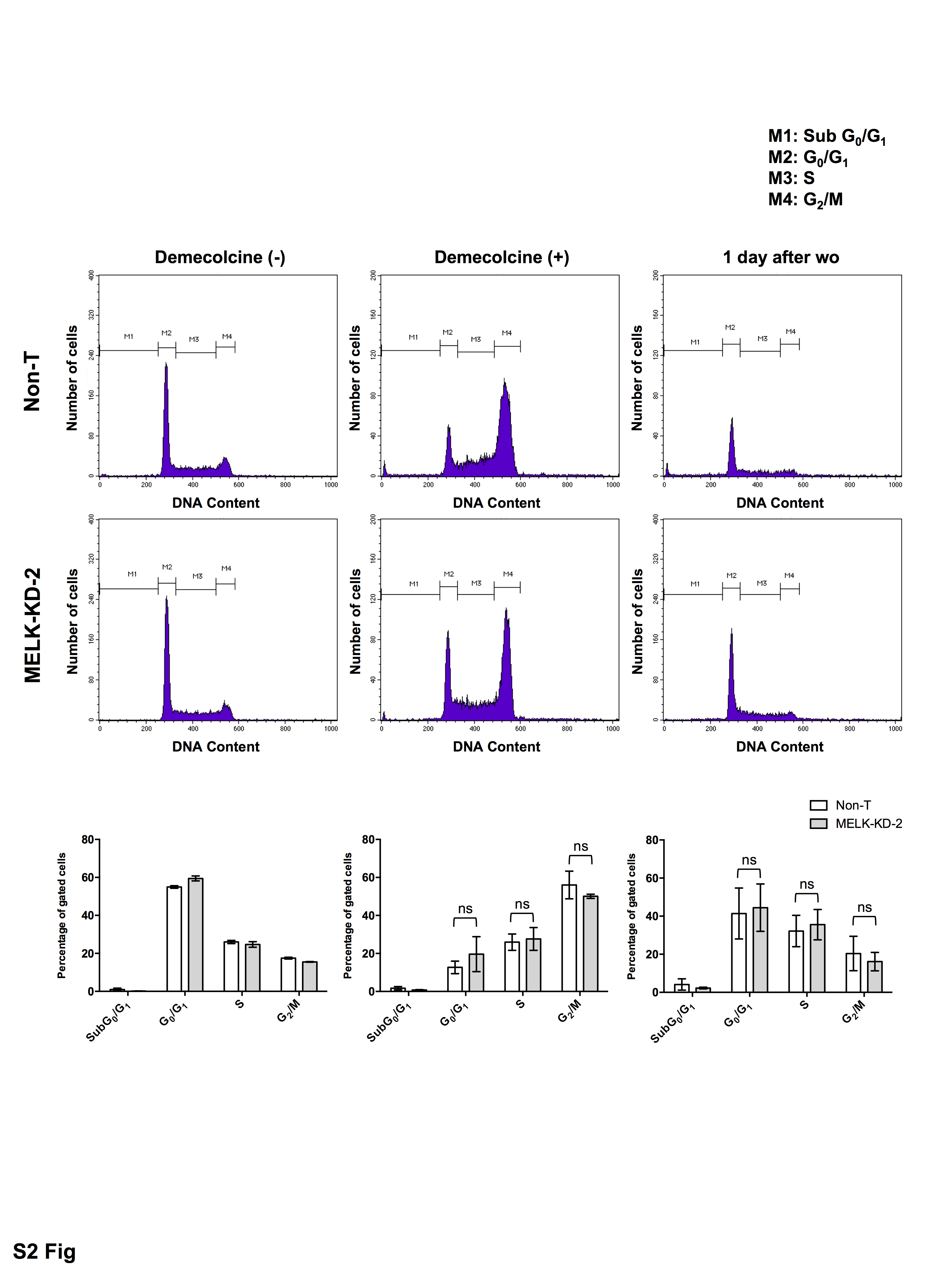

Supplement: S2 Fig — Effects of MELK depletion on cell cycle progression were determined using propidium iodide (PI) staining and FACS analysis. Non-T and MELK-KD-2 MT4C5 cells were treated with [top and middle panels Demecolcine (+), Non-T and MELK-KD-2] or without [top and middle panels Demecolcine (-), Non-T and MELK-KD-2] 0.05 μg/ml Demecolcine (Wako Pure Chemical Industries, Ltd., Osaka, Japan) for 16 h to synchronize them in M phase. Demecolcine was then removed and the cells were cultured for 24 h with fresh growth medium, stained with PI, and analyzed by FACS [top and middle panels 1 day after washout (wo), Non-T and MELK-KD-2]. Graphs show the distribution of cells in distinct cell cycle phases from five independent experiments (bottom panels). The average percentages of cells in each cell cycle phase (SubG0/G1, G0/G1, S and G2/M) are shown [bottom left panel: Demecolcine (-), bottom middle panel: Demecolcine (+), bottom right panel: 1 day after wo]. Error bars are standard deviations calculated from five independent experiments. Statistical significance was determined by unpaired two-tailed Student’s t test. ns, not significant (P>0.05). (TIFF) [file ppat.1006441.s004.tiff]

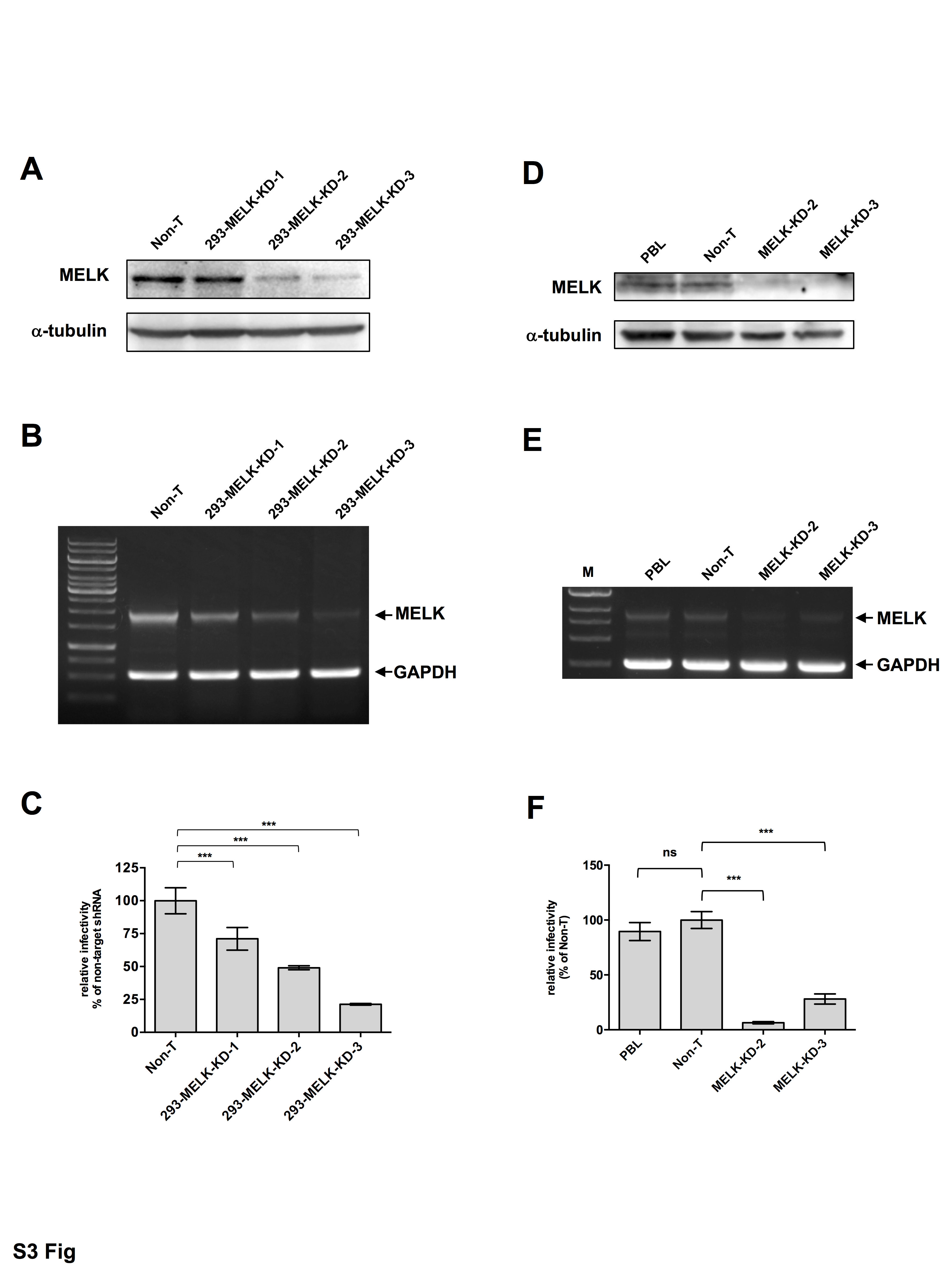

Supplement: S3 Fig — (A) Lysates of HEK293 cells stably expressing non-target shRNA or MELK-specific shRNA (MELK-KD-1, 2 and 3) were immunoblotted with anti-MELK or anti-alpha-tubulin antibodies. (B) Total RNA from the cells in (A) was extracted and examined for MELK mRNA expression by multiplex RT-PCR amplification (MELK). The primer set for amplification of GAPDH mRNA was included in each reaction as an internal control (GAPDH). (C) Effect of MELK depletion on single-round HIV-1 infection in HEK293 cells. HEK293 cells described in (A) and (B) were infected with VSV-G-pseudotyped NL4-3luc. The mean luciferase value from non-target shRNA HEK293 cells was arbitrarily set as 100%. Error bars reflect the standard deviations calculated from five independent experiments. (D) Lysates of CD3/CD28-stimulated PBLs stably expressing non-target shRNA or MELK-specific shRNA (PBL-MELK-KD-2 and 3) were immunoblotted with anti-MELK or anti-alpha-tubulin antibodies. (E) Total RNA was extracted and MELK mRNA expression determined by multiplex RT-PCR amplification (MELK). A primer set for amplification of GAPDH mRNA was included in each reaction as an internal control (GAPDH). (F) Effect of MELK depletion on a single-round of HIV-1 infection in CD3/CD28-stimulated PBL. PBL, Non-T, PBL-MELK-KD-2 and PBL-MELK-KD-3 cells described in (D) and (E) were infected with VSV-G-pseudotyped NL4-3luc. The mean luciferase value from non-target shRNA CD3/CD28-stimulated PBL was arbitrarily set as 100%. Error bars are standard deviations calculated from five independent experiments. Statistical significance was determined by one-way analysis of variance (ANOVA) with Dunnett’s multiple comparison test (C and F). ns, not significant (P>0.05); *P<0.05, **P<0.01, ***P<0.001. (TIFF) [file ppat.1006441.s005.tiff]

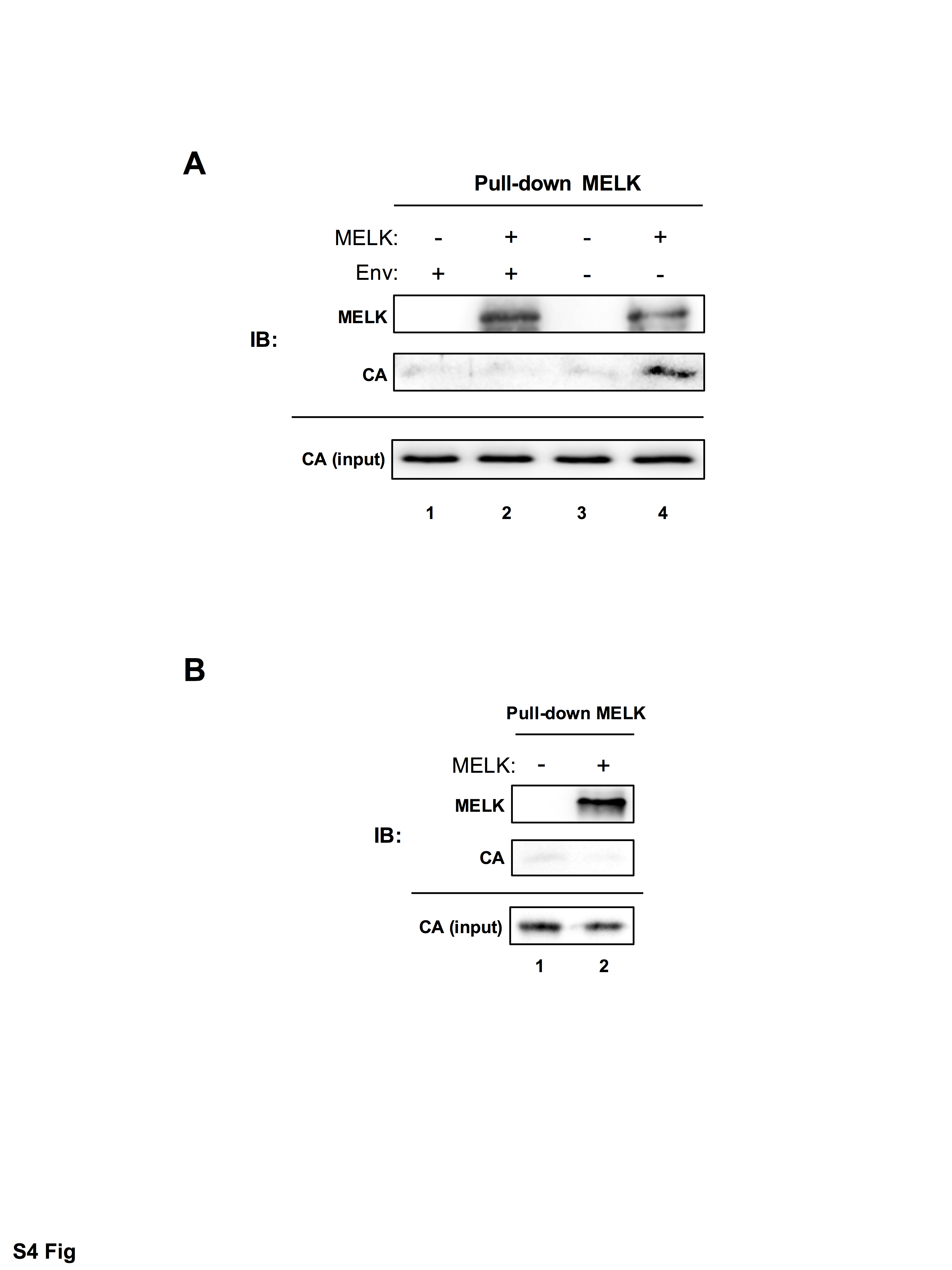

Supplement: S4 Fig — (A) HeLa cells were transfected with pCAG-OSF or pCAG-OSF-MELK. Transfected HeLa cell extracts were incubated with Strep-Tactin Sepharose and OSF-tagged proteins were purified. OSF-tagged control (lanes 1 and 3) and MELK (lanes 2 and 4) proteins were then incubated with purified HIV-1 virions (lanes 1 and 2) or envelope-stripped cores (lanes 3 and 4), and complex formation was assessed by immunoblotting (IB) using rabbit anti-MELK antibody (MELK) and mouse anti-p24 antibody (CA). (B) Purified OSF-tagged control (lane 1) and MELK (lane 2) proteins were incubated with soluble CA (input). Complex formation was assessed by immunoblotting (IB) using rabbit anti-MELK antibody (MELK) and mouse anti-p24 antibody (CA). (TIFF) [file ppat.1006441.s006.tiff]

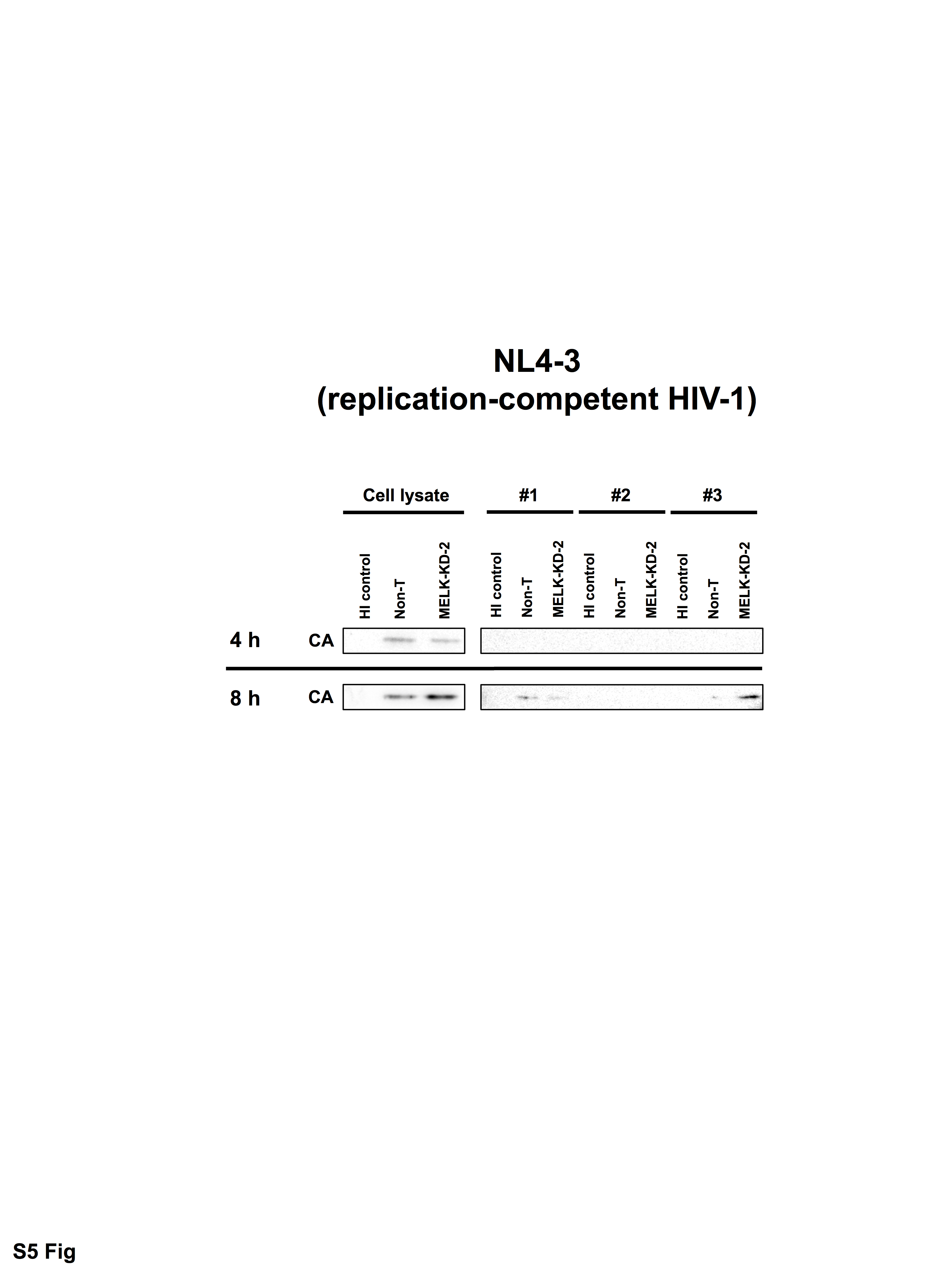

Supplement: S5 Fig — Effect of MELK depletion on the fate of the HIV-1 CA in MT4C5 cells at 4 and 8 h post-infection analyzed as in Fig 2I. (TIFF) [file ppat.1006441.s007.tiff]

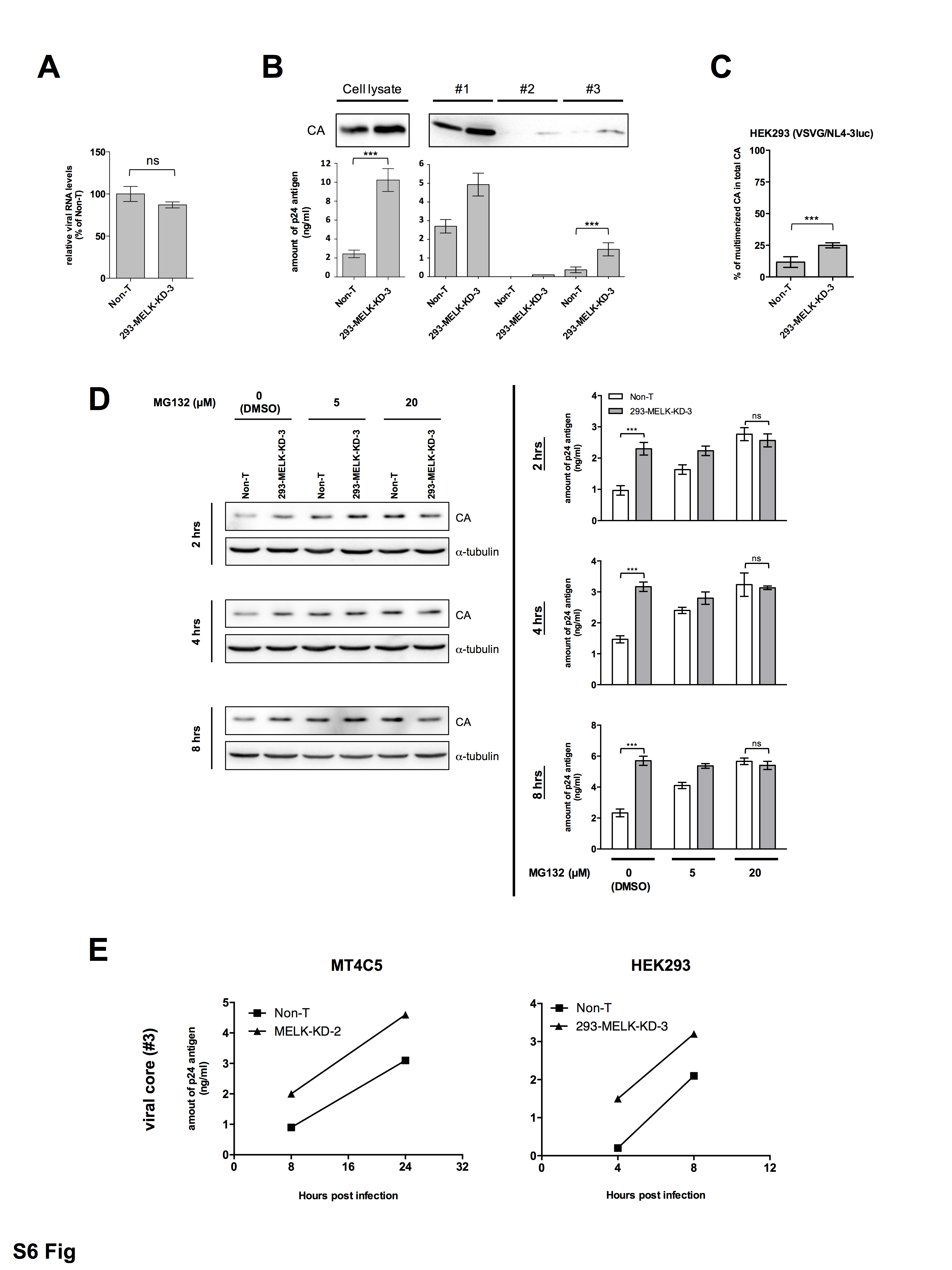

Supplement: S6 Fig — (A) Virion-associated viral RNA was quantified by quantitative RT-PCR 2 h after infection of Non-T or 293-MELK-KD-3 cells with wild-type HIV-1. (B) Effect of MELK depletion on the fate of the HIV-1 CA in HEK293 cells analyzed as in Fig 2I. (C) Percentage of the pelletable CA within total CA as quantified by p24 ELISA shown in S6B Fig. (D) Non-T or MELK-KD-3 HEK293 cells were infected with VSV-G-pseudotyped HIV-1 for 2, 4 or 8 h in the presence or absence of the proteasome inhibitor MG132 (0, 5, or 20 μM). Whole cell lysates were immunoblotted with anti-p24 (CA) or anti-alpha-tubulin (α-tubulin) antibodies (left panels). Experiments were performed five times and one representative set of data is shown. The amounts of CA in the whole cell lysates were quantified by HIV-1 p24 ELISA (right panels). Error bars indicate the standard deviations calculated from five independent experiments. (E) Results of p24 ELISA showing the steady-state levels of CA in fraction #3 in Fig 2I and S6B Fig at the indicated time points (MT4C5, left panel; HEK293, right panel). Statistical significance was determined by two-way analysis of variance (ANOVA) with Sidak’s multiple comparison test (D), or unpaired two-tailed Student’s t test (A, B, and C). ns, not significant (P>0.05); *P<0.05, **P<0.01, ***P<0.001. (TIFF) [file ppat.1006441.s008.tiff]

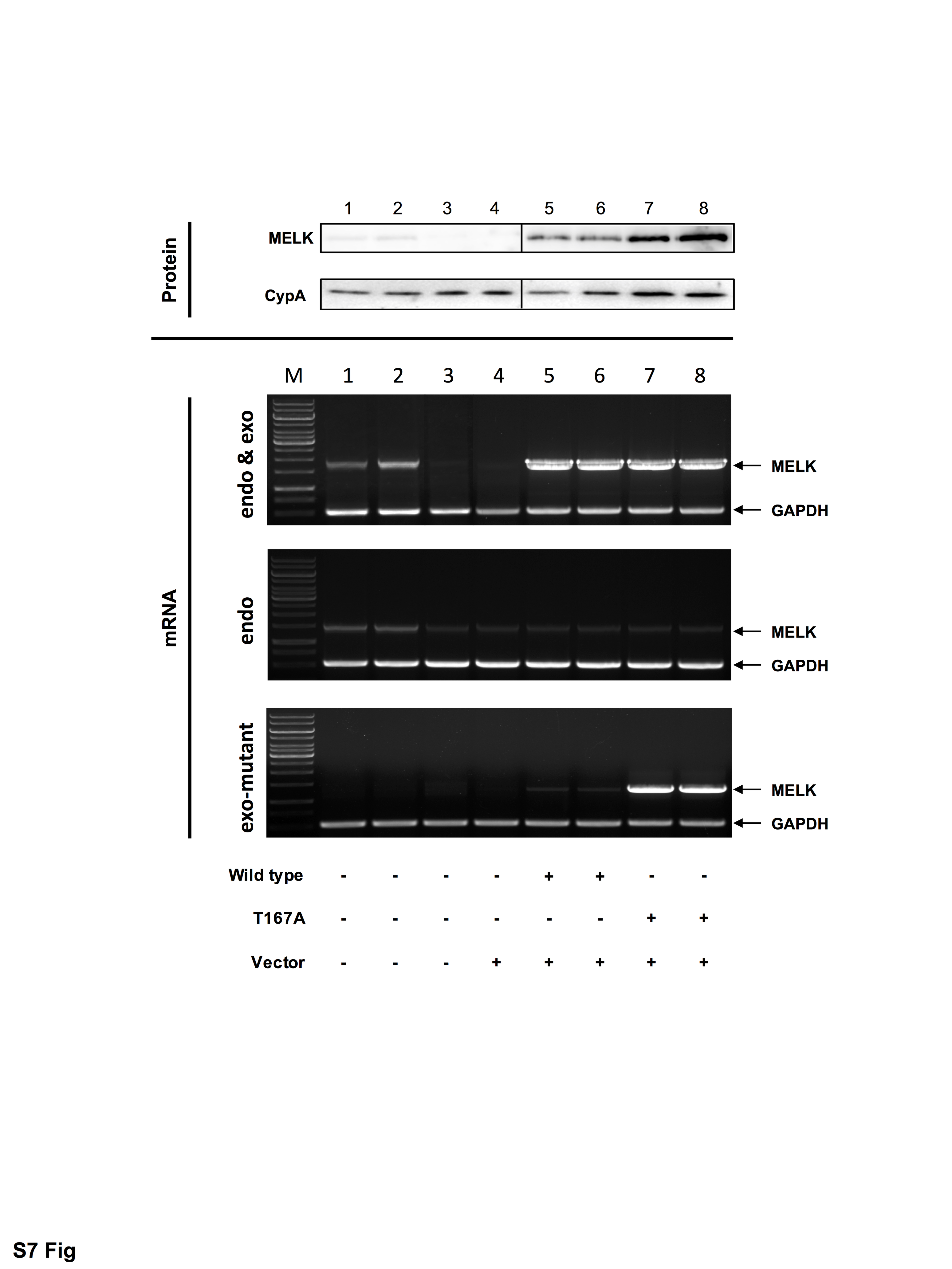

Supplement: S7 Fig — Panel protein: Parental MT4C5 (lane 1), Non-T (lane 2) or MT4C5-MELK-KD-1 cells (lanes 3) transduced with control vector (lane 4) or vector for wild-type MELK (lanes 5 and 6, two independent cell pools) or catalytically inactive T167A MELK mutant (lanes 7 and 8, two independent cell pools) were used. Cell lysates were immunoblotted with anti-MELK or anti-α-tubulin antibodies. Panel mRNA: Total RNA from cells listed above was extracted. Total MELK mRNAs (upper panel), endogenous MELK mRNA (middle panel) and exogenous mutant MELK mRNA (bottom panel) were quantified by RT-PCR amplification with specific primer sets (MELK). The primer set for amplification of GAPDH mRNA was included in each reaction as an internal control (GAPDH). Experiments were performed three times and one set of representative data is shown. (TIF) [file ppat.1006441.s009.tif]

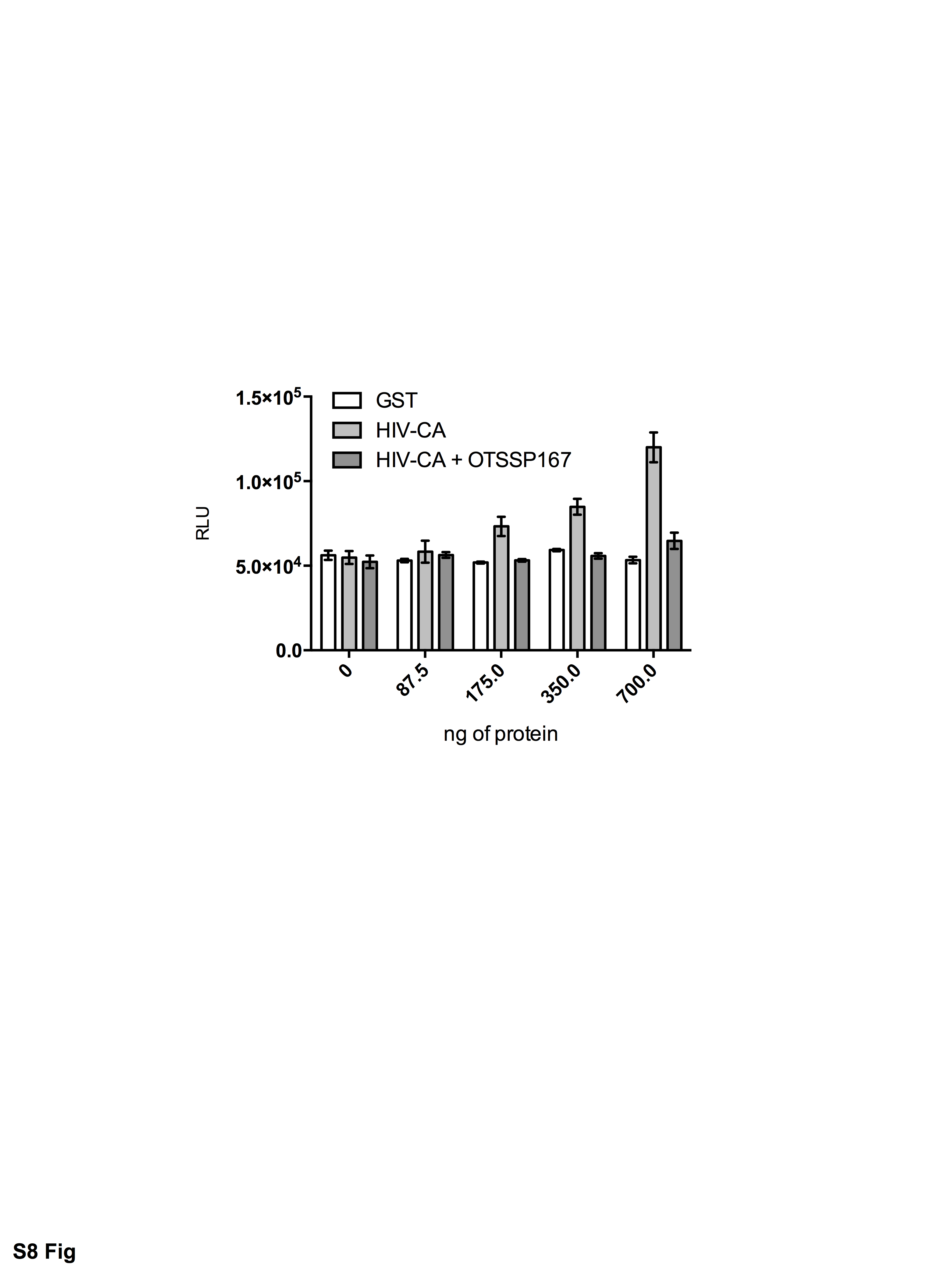

Supplement: S8 Fig — Phosphorylation of recombinant CA by MELK was monitored as in Fig 3C. Error bars reflect the standard deviations calculated from three independent experiments. (TIFF) [file ppat.1006441.s010.tiff]

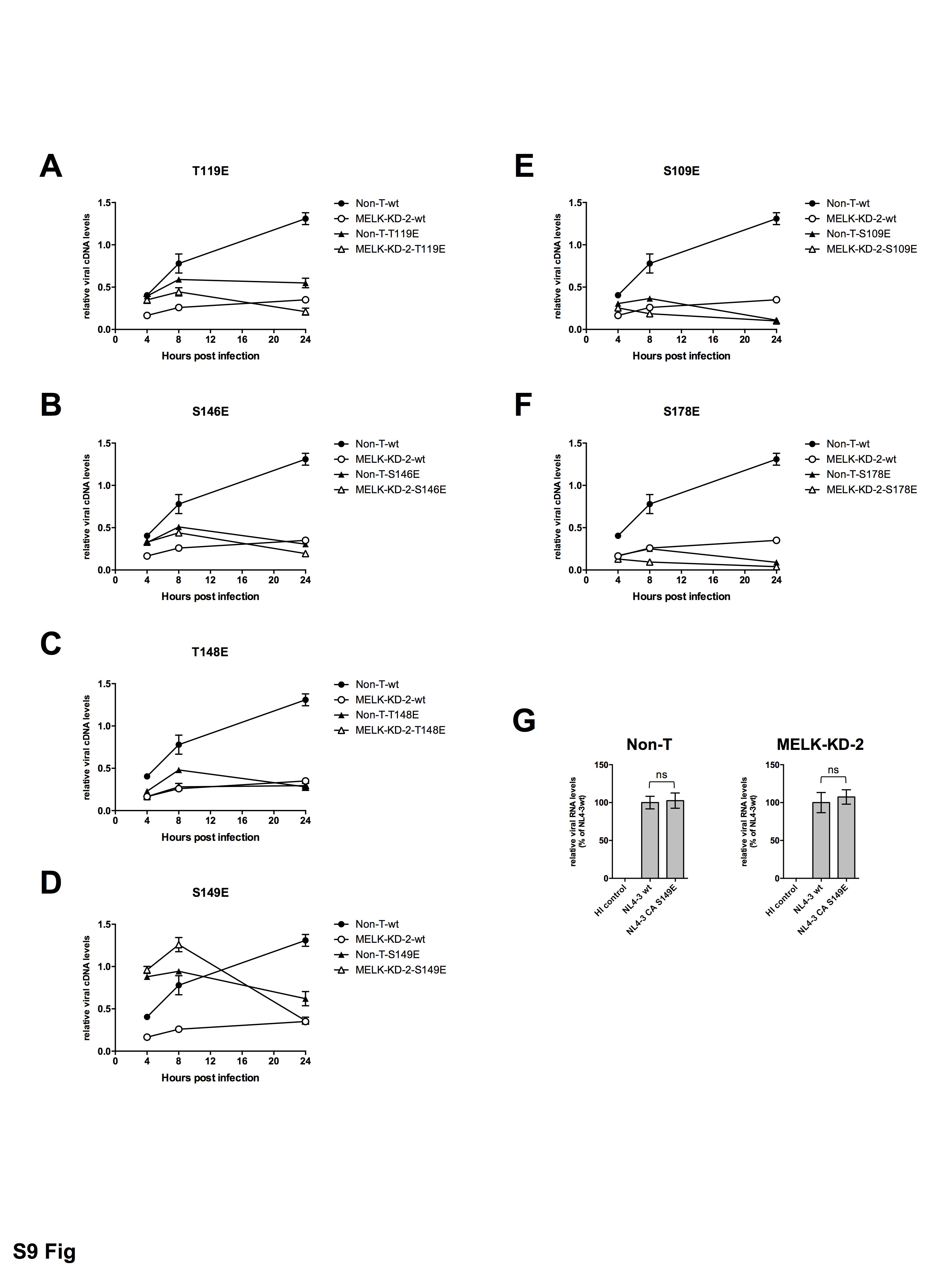

Supplement: S9 Fig — (A-F) Total DNA was extracted from non-target shRNA (Non-T) or MELK-depleted (MELK-KD-2) MT4C5 cells at the indicated time points (4, 8 and 24 h) after wild-type or indicated mutants of HIV-1 infection and analyzed for the amounts of late RT product containing the env region. Experiments were performed at least three times and error bars are standard deviations calculated from three independent experiments. The ratios of each viral cDNA level to beta-globin DNA level are given. (G) Quantitative RT-PCR analyses of virion-associated viral RNA at 2 h after infection of Non-T or MELK-KD-2 MT4C5 cells with wild-type HIV-1 or CA S149E HIV-1 mutant. Error bars indicate the standard deviations calculated from five independent experiments. Statistical significance was determined by unpaired two-tailed Student’s t test (G). ns, not significant (P>0.05); *P<0.05, **P<0.01, ***P<0.001. (TIFF) [file ppat.1006441.s011.tiff]

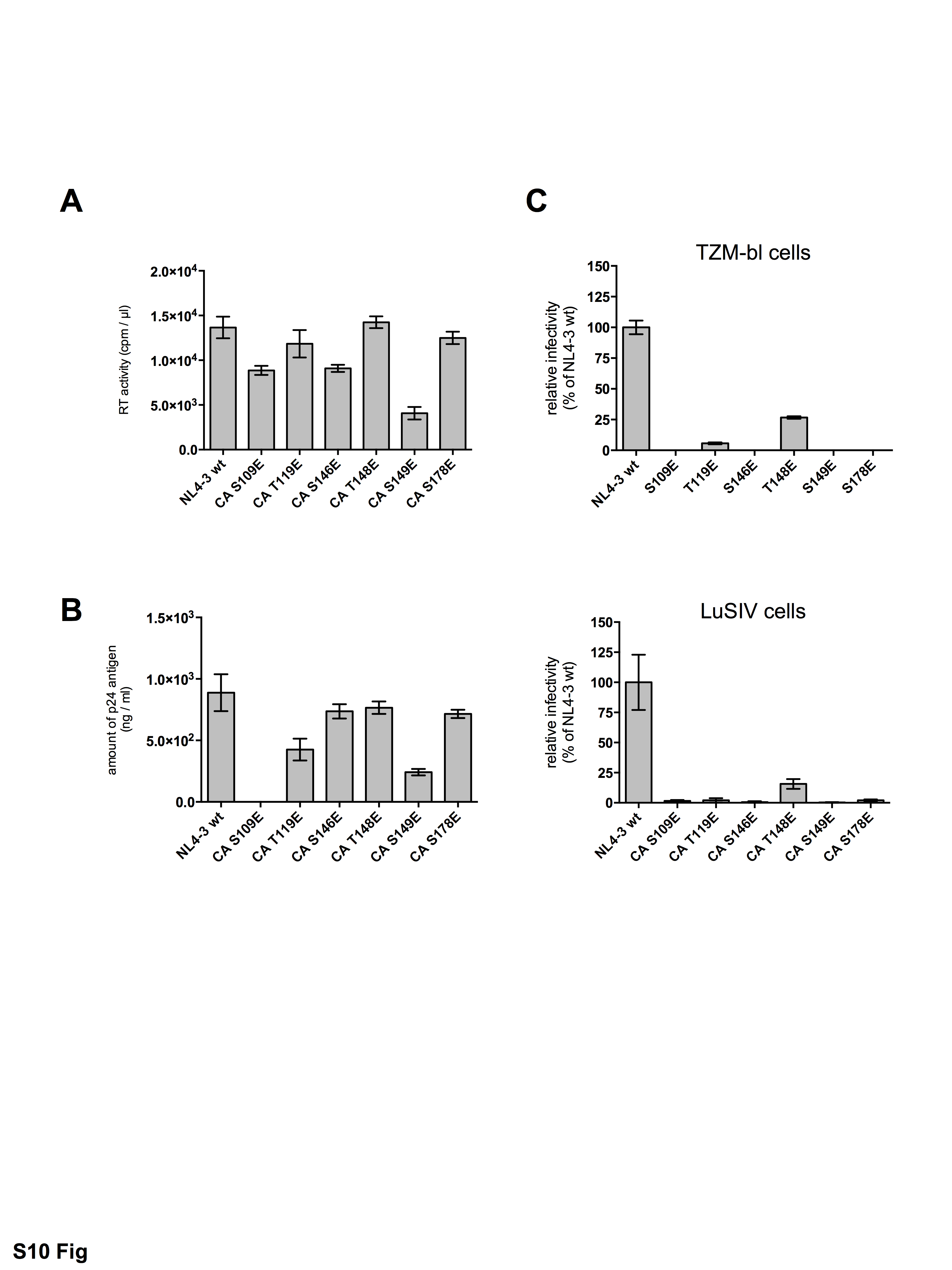

Supplement: S10 Fig — (A, B) Virus production was monitored by assessing RT activity (A) or p24 antigen (B) in culture supernatants of HeLa cells. (C) Viral infectivity was evaluated by infection of TZM-bl (upper panel) and LuSIV (lower panel) indicator cell lines with culture supernatants shown in (A) normalized by RT activity. Relative luciferase activities are shown as percentages (%) of that of NL4-3wt with standard deviations calculated from five independent experiments. (TIFF) [file ppat.1006441.s012.tiff]

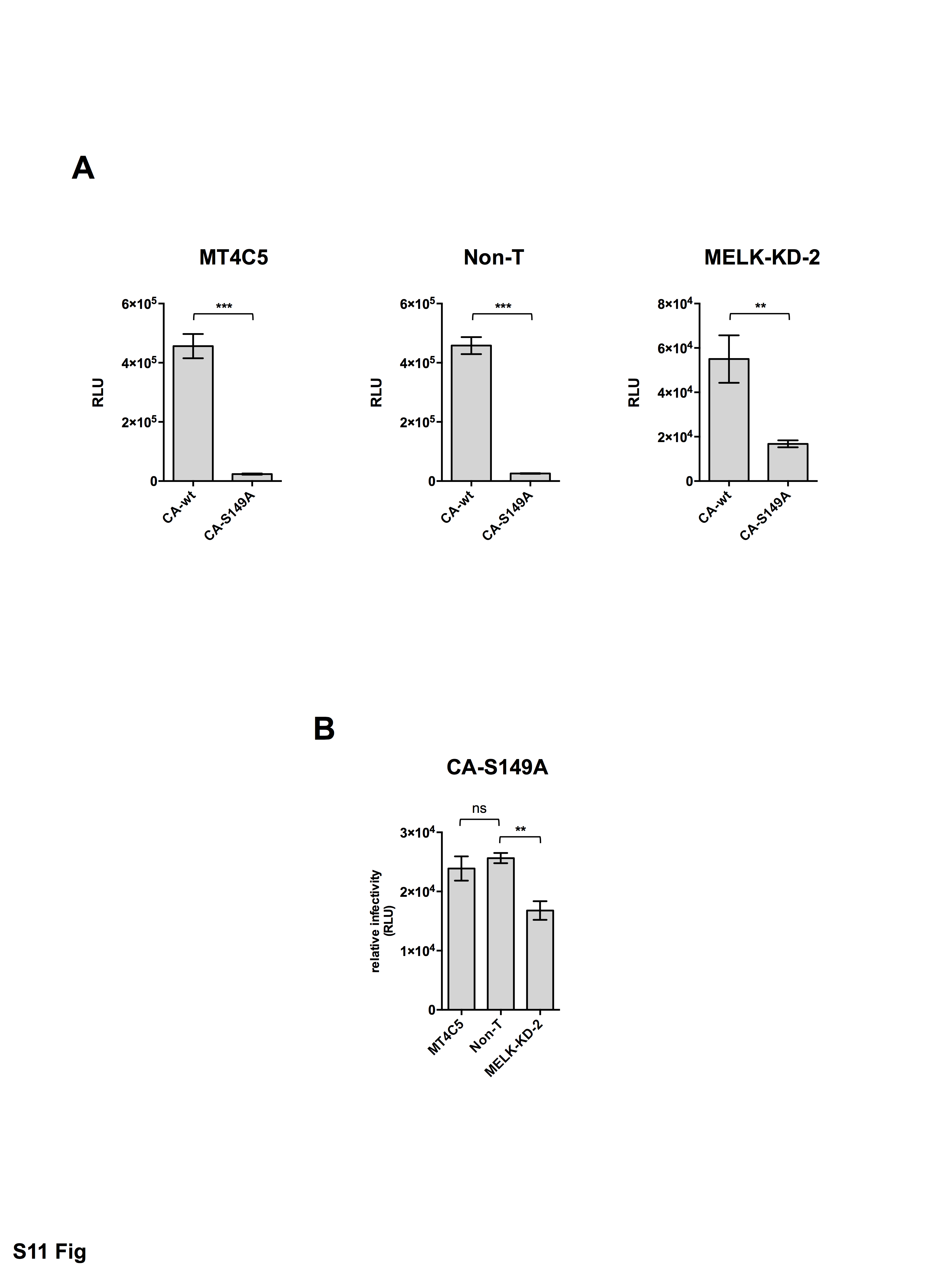

Supplement: S11 Fig — (A) MT4C5, Non-T and MELK-KD-2 cells were infected with VSV-G-env-pseudotyped NL4-3luc CA-wt or CA-S149A normalized by reverse transcriptase (RT) counts corresponding to 10 ng (p24) of VSV-G/NL4-3luc CA-wt. Relative luciferase activities are shown with standard deviations calculated from five independent experiments. (B) The CA-S149A results in each cell pool were compared on the same Y axis setting. Statistical significance was determined by unpaired two-tailed Student’s t test (A), or one-way analysis of variance (ANOVA) with Dunnett’s multiple comparison test (B). ns, not significant (P>0.05); *P<0.05, **P<0.01, ***P<0.001. (TIFF) [file ppat.1006441.s013.tiff]

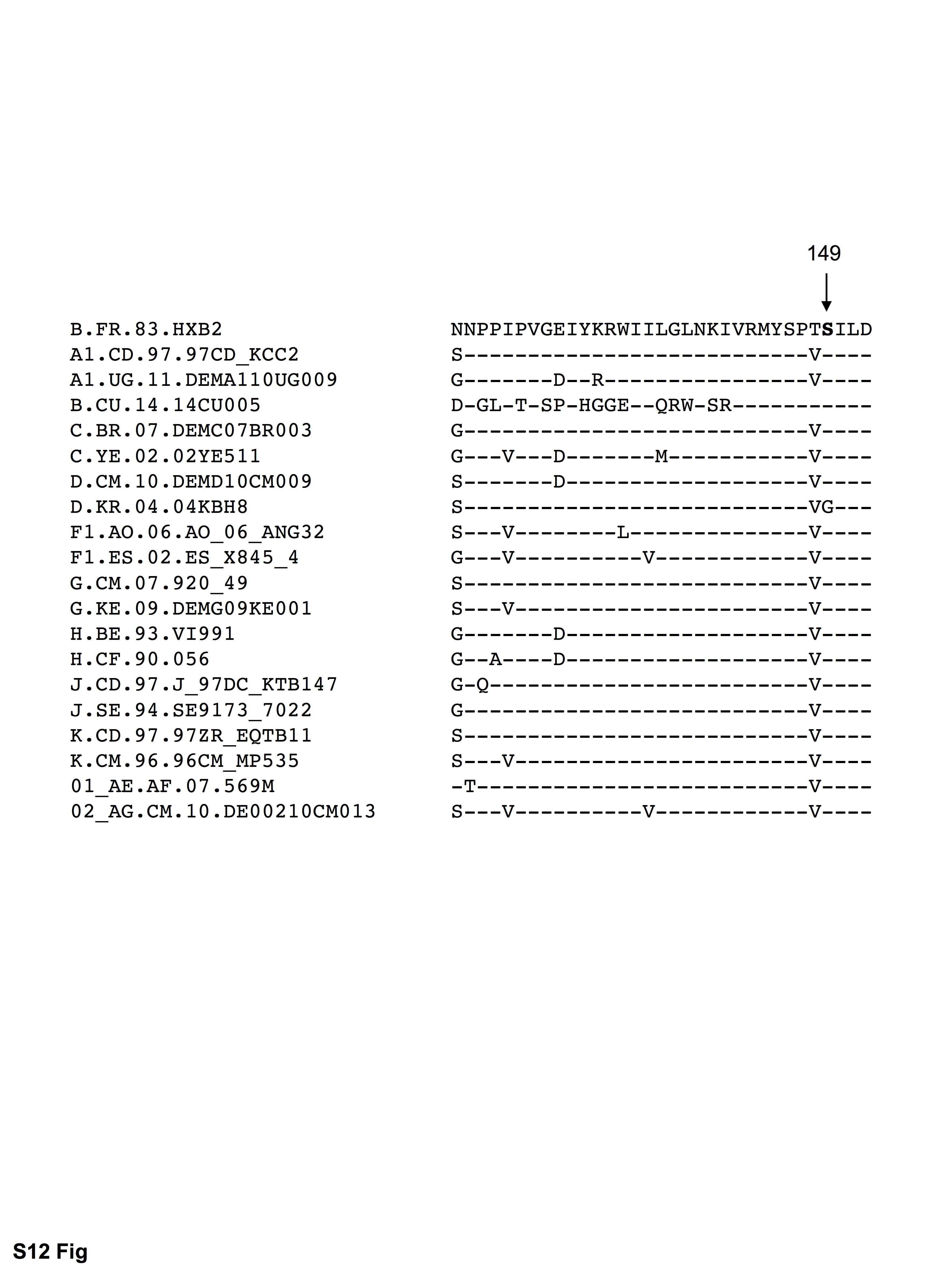

Supplement: S12 Fig — The sequences are aligned with the HIV.HXB2 sequence. The arrow indicates the 149th amino acid from the N-terminus of HIV-1 capsid. Dashes indicate amino acid sequence identity. (TIFF) [file ppat.1006441.s014.tiff]

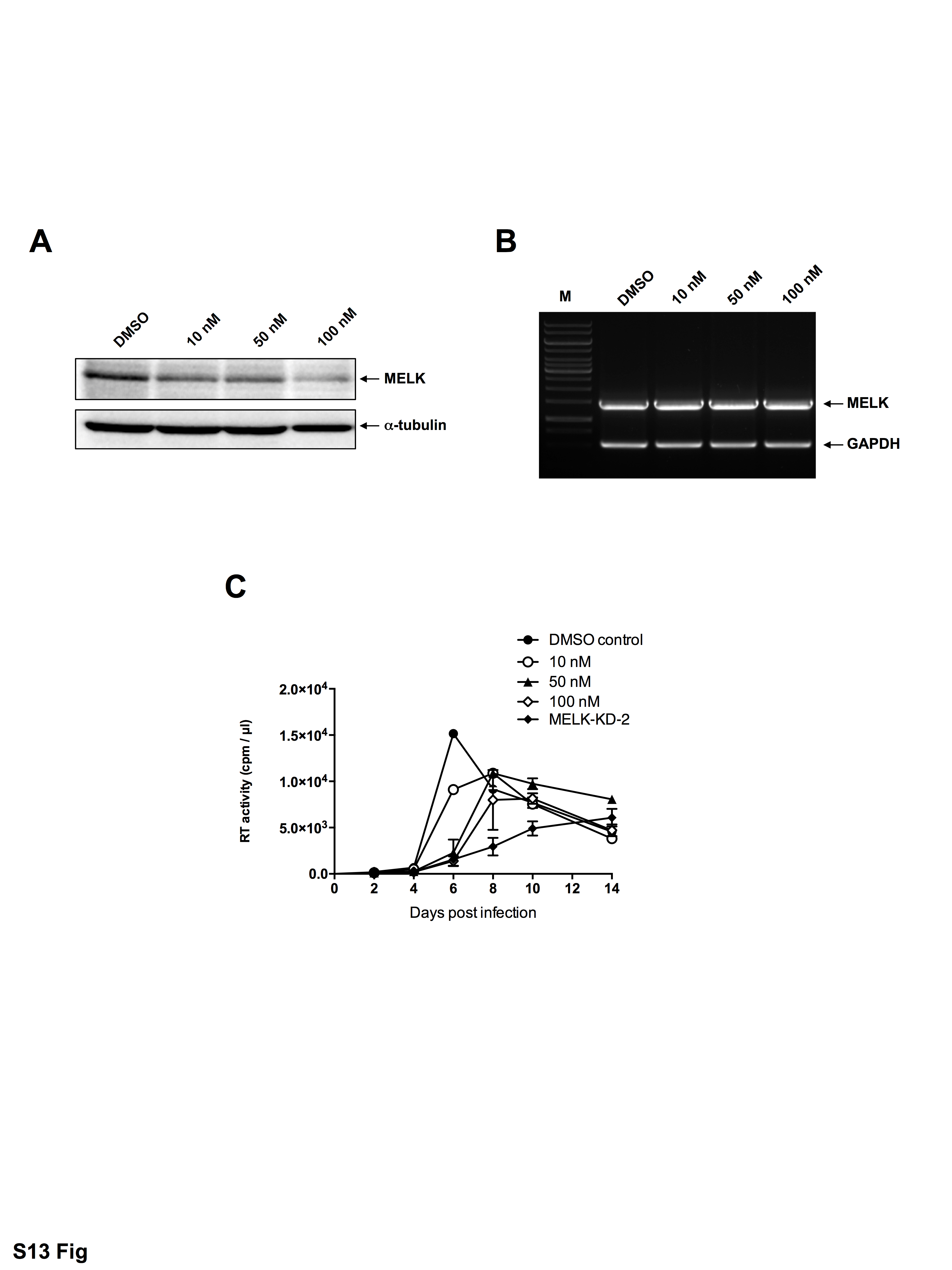

Supplement: S13 Fig — (A) Immunoblot analyses with anti-MELK (upper panel) or α-tubulin (lower panel) monitoring endogenous MELK expression in MT4C5 cells treated with increasing amounts of Siomycin A. (B) Semi-quantitative RT-PCR analysis of MELK and GAPDH mRNA expression in MT4C5 cells described in (A). (C) Effect of Siomycin A on HIV-1 replication in MT4C5 cells. The virion-associated RT activity was monitored at the indicated time points in culture supernatants of MT4C5 cells treated with Siomycin A (10 nM: open circles, 50 nM: closed triangles, 100 nM: open diamonds) and those of MELK-KD-2 (closed diamonds). Error bars reflect the standard deviations calculated from three independent experiments. (TIFF) [file ppat.1006441.s015.tiff]
